# Targeting immune checkpoints in hepatocellular carcinoma therapy: toward combination strategies with curative potential

**DOI:** 10.1186/s40164-025-00636-5

**Published:** 2025-05-02

**Authors:** Jing Tong, Yongci Tan, Wenwen Ouyang, Haocai Chang

**Affiliations:** 1 MOE Key Laboratory of Laser Life Science & Institute of Laser Life Science, College of Biophotonics, School of Optoelectronic Science and Engineering, South China Normal University, Guangzhou, 510631 China; 2 Guangdong Provincial Key Laboratory of Laser Life Science, College of Biophotonics, School of Optoelectronic Science and Engineering, South China Normal University, Guangzhou, 510631 China

**Keywords:** Hepatocellular carcinoma, Immune checkpoint inhibitors, ICI monotherapy, Dual ICI therapy, ICI-based combination therapy

## Abstract

Hepatocellular carcinoma (HCC) is a primary liver cancer characterized by poor immune cell infiltration and a strongly immunosuppressive microenvironment. Traditional treatments have often yielded unsatisfactory outcomes due to the insidious onset of the disease. Encouragingly, the introduction of immune checkpoint inhibitors (ICIs) has significantly transformed the approach to HCC treatment. Moreover, combining ICIs with other therapies or novel materials is considered the most promising opportunity in HCC, with some of these combinations already being evaluated in large-scale clinical trials. Unfortunately, most clinical trials fail to meet their endpoints, and the few successful ones also face challenges. This indicates that the potential of ICIs in HCC treatment remains underutilized, prompting a reevaluation of this promising therapy. Therefore, this article provides a review of the role of immune checkpoints in cancer treatment, the research progress of ICIs and their combination application in the treatment of HCC, aiming to open up avenues for the development of safer and more efficient immune checkpoint-related strategies for HCC treatment.

**Graphical abstract:**

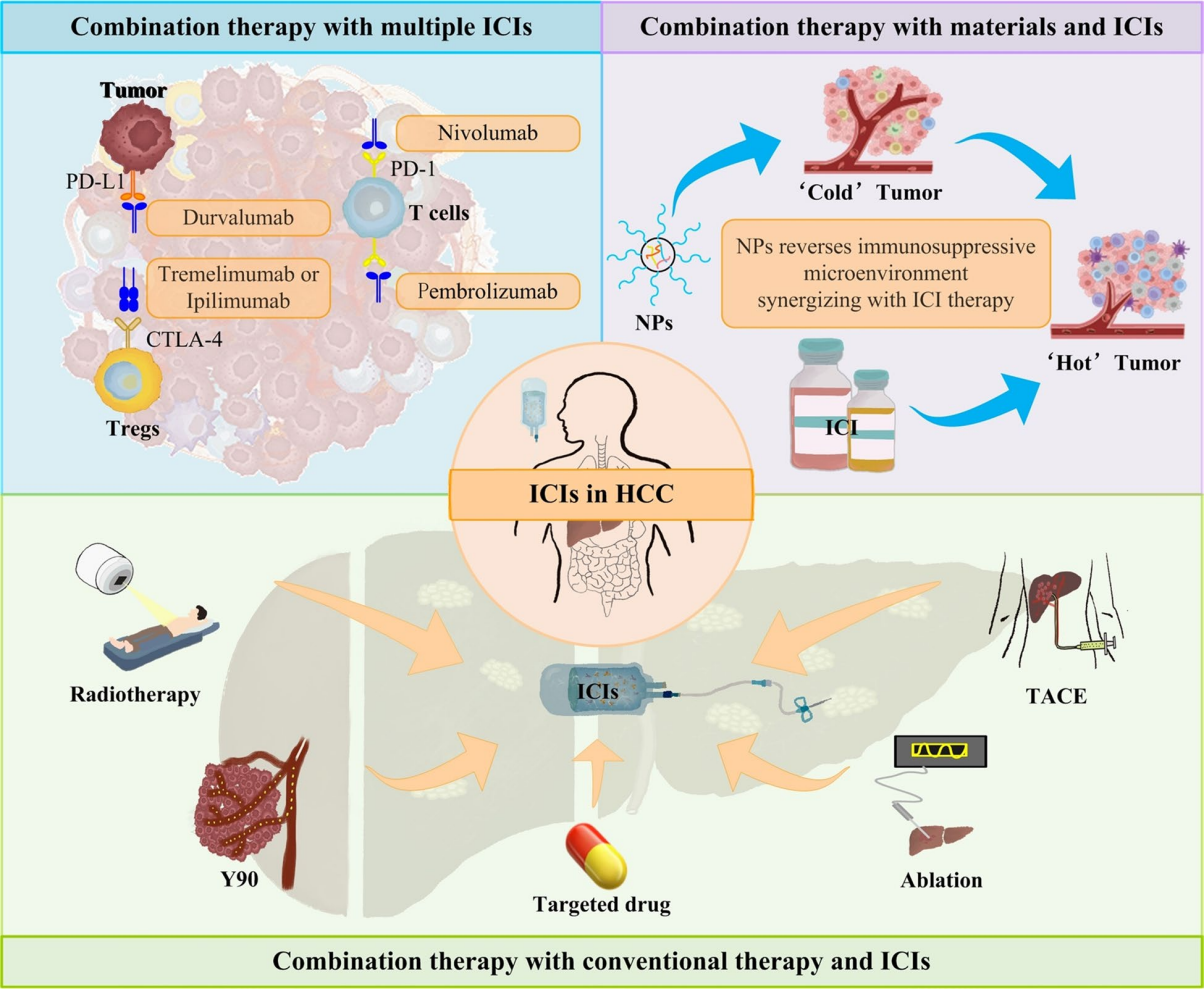

## Introduction

Hepatocellular carcinoma (HCC) is the most common type of liver cancer, representing 75–85% of all cases [1]. It is a primary liver cancer with well-known major risk factors including excessive alcohol consumption, hepatitis B and C infections, diabetes, and metabolic diseases [2]. Clinically, the main treatments for liver cancer are surgical resection, liver transplantation, and local ablation [3, 4]. However, the majority of liver cancer patients have an insidious disease onset, making early diagnosis challenging [5]. Most patients are diagnosed at a late stage or after metastasis has occurred, missing the opportunity for curative treatment. The disease progresses rapidly with a poor prognosis, high mortality rate, and even with radiation or chemotherapy, the outcomes remain suboptimal due to resistance and recurrence, leading to a low survival rate [6]. Despite efforts to promote early screening in high-risk populations, over 80% of patients are diagnosed at a late stage with inoperable disease, and current treatment strategies fall short in preventing high rates of metastasis and recurrence in HCC patients [7]. HCC is a solid tumor with complex pathophysiological barriers, poor immune cell infiltration, and a strong tumor immunosuppressive microenvironment, all of which greatly limit the effectiveness of immunotherapy [1]. Therefore, further optimization of the tumor immune microenvironment in HCC is crucial for enhancing the efficacy of immunotherapy in HCC patients. In recent years, there have been breakthroughs in systemic treatments for HCC. Systemic pharmacotherapy mainly consists of molecular-targeted drugs and immunotherapy [8]. Immune checkpoint inhibitors (ICIs) represent a pivotal systemic treatment modality that has achieved considerable success in numerous malignant tumors and is extensively employed in the management of HCC [9]. ICIs modulate immune responses by interacting with ligands of immune checkpoints on peripheral tissues and on T cells, thereby minimizing collateral damage to healthy tissues [10]. Clinical studies have demonstrated that immune checkpoint inhibitor therapy prolongs patient survival with minimal adverse reactions, making it a viable adjunctive treatment alongside surgery, radiotherapy, and chemotherapy [11]. However, due to the complex pathogenesis of HCC and the immunosuppressive tumor microenvironment (TME), the response rate to monotherapy with ICIs in HCC is less than 20% [12], while the efficacy of combination therapy is approximately 30–36% [13]. Combination therapy represents a novel treatment strategy for patients developing resistance to conventional treatment modalities [14]. These combination regimens, centered around ICIs, primarily involve combination with other ICIs, targeted therapies, chemotherapy, and local therapies [13]. This paper provides a comprehensive review of the role of immune checkpoints in cancer therapy, particularly focusing on immune therapy based on ICIs in HCC, with emphasis on the research progress of ICIs combined with other treatment modalities for HCC.

## The immune checkpoints in HCC

### Types of immune checkpoints

Immune checkpoints play pivotal regulatory roles within the immune system, primarily modulating the intensity and duration of immune responses to ensure timely activation or inhibition [15]. These checkpoints typically consist of protein molecules on the cell surface, which regulate the activity of immune cells through interactions or signaling pathways [16]. Such regulatory mechanisms provide essential support for the balance of the immune system, enabling it to combat both exogenous and endogenous threats and maintain immune homeostasis [17, 18]. Therefore, research and understanding of immune checkpoints are of paramount importance in the development of immunomodulatory therapies and combating immune-related diseases.

#### Classical immune checkpoints

**Fig. 1:**
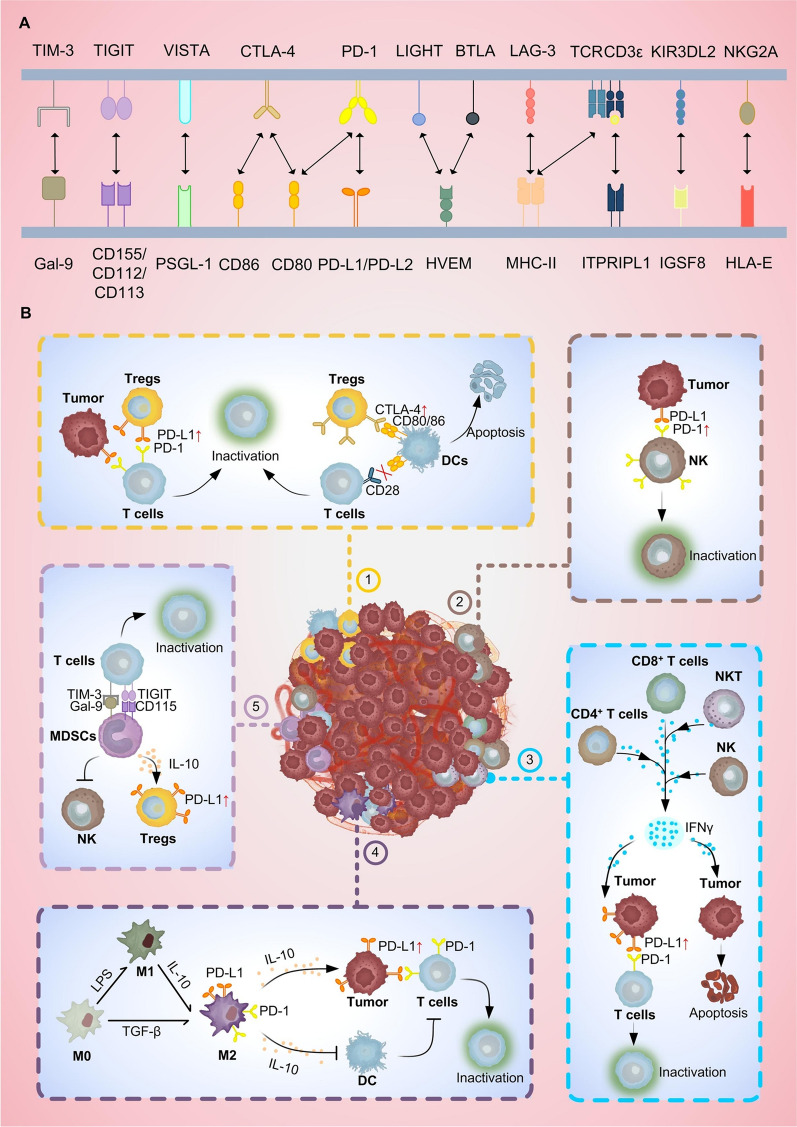
The role of immune checkpoints in the TME. **A** Ligands and their receptors of immune checkpoints. **B** The role of immune checkpoints in the TME. (1) Tregs overexpressed immune checkpoint PD-L1 and CTLA-4, accumulating in large quantities in the TME, where they can deplete T cells. Specifically, PD-L1 expressed on Tregs and tumor cells binds to PD-1 on the surface of T cells, causing T cell inactivation; CTLA-4 expressed on Tregs preferentially bind to APCs (CD80 and CD86), depriving T cells of stimulative signals; Moreover, they have the capability to induce apoptosis of DCs and eventually lead to reduced immune response. (2) The expression of PD-1 is upregulated in tumor-infiltrating NK cells, which induce NK cell apoptosis through PD-L1 on tumor cells. (3) CD4^+^ T cells, CD8^+^ T cells, NK cells, and NKT cells produce IFN-γ, which induces apoptosis or non-apoptotic cell death in most tumor environments. However, when tumor cells are chronically exposed to IFN-γ, it induces PD-L1 upregulation and then immune escape [75]; In another scenario, the expression of PD-L1 on ovarian tumor cells has been observed to be strongly induced by IFN-γ, leading to T cell inactivation [212]. (4) TAM polarizes into M2-like macrophages, which upregulates the expression of PD-1 and PD-L1. M2 secretes IL-10, and then upregulates the expression of PD-L1 in tumor cells and downregulates the stimulation ability of DCs on T cells, finally exhausts T cells. (5) MDSCs inhibit T cells by binding to immune checkpoints TIM-3 and TIGIT, while also suppressing NK cell-mediated anti-tumor immune response. In addition, IL-10 secreted by MDSCs also induces the upregulation of PD-L1 in Tregs, thereby inhibiting the immune response

1. PD-1/PD-L1 axis
  The PD-1/PD-L1 axis plays a crucial role in immune regulation (Fig. 1A). PD-1 is one of the most important co-inhibitory receptors expressed on the surface of T cells, which belongs to the CD28 immunoglobulin superfamily [19]. It is transiently expressed during the activation of various immune cells, including T cells, B cells, natural killer (NK) cells, natural killer T (NKT) cells, macrophages, and dendritic cells (DCs). PD-1 exerts negative immune regulatory functions by binding to its ligands, PD-L1 or PD-L2 [20]. T cell activation relies on two signals: the first signal is generated by the interaction between the T cell receptor (TCR) and the major histocompatibility complex (MHC)-antigen peptide complex on antigen-presenting cells (APCs), which is responsible for antigen-specific recognition. The second signal is provided by the co-stimulatory molecules CD80/CD86 on APCs binding to the co-stimulatory receptor CD28 on T cells, which is essential for full activation. Only when both signals are present can T cells achieve complete activation and exert their effector functions [21]. At the molecular level, when PD-1 binds to its ligand, it triggers tyrosine phosphorylation of PD-1 and recruits SHP2, leading to the widespread dephosphorylation of T cell activation kinases. This process not only attenuates the signaling of the TCR and CD28 pathways but also further suppresses immune responses [22]. In the TME, cancer cells evade immune surveillance by expressing PD-L1 or PD-L2, which bind to PD-1 on T cells, thereby promoting tumor progression [23]. Notably, the interaction between PD-1 and PD-L1 also influences the formation of memory T cells, thereby regulating the persistence and durability of immune responses [24].
2. CTLA-4/CD80 (CD86) axis
  CTLA-4 is a transmembrane receptor on T cells and is mainly expressed on B lymphocytes and activated CD8^+^ and CD4^+^ T lymphocyte surfaces. Studies have demonstrated that CTLA-4 plays a crucial inhibitory role in immune regulation through multiple mechanisms, including suppression of T cell proliferation, modulation of cytokine expression, and regulation of APCs. The CD28 molecule on T cells interacts with its ligands, CD80 and CD86, on APCs such as DCs, triggering a co-stimulatory signal that promotes T cell activation and enhances immune responses. However, since CD28 exhibits a lower binding affinity for CD86 monomers compared to CTLA-4, and CTLA-4 has a significantly higher affinity for CD80/CD86, it can outcompete CD28 for ligand binding, thereby inhibiting CD28-mediated T cell activation [25]. Beyond its role in blocking CD28 signaling, CTLA-4 also modulates APC function through reverse signaling via CD80/CD86, further impairing the antigen presentation process. Upon binding to CD80/CD86, CTLA-4 induces the expression of indoleamine 2,3-dioxygenase (IDO) in APCs, leading to the catabolism of tryptophan. This metabolic shift depletes a crucial nutrient required for T cell proliferation, thereby suppressing T cell responses [26]. Additionally, CTLA-4 engagement with CD80 can trigger trans-endocytosis, whereby CD80 is actively removed from the APC surface. This reduces the availability of CD80 for CD28 interaction, further limiting the ability of APCs to activate T cells [27]. Moreover, CTLA-4 signaling can drive the secretion of immunosuppressive cytokines, such as transforming growth factor-β (TGF-β), while simultaneously downregulating the production of stimulatory cytokines like Interleukin-2 (IL-2), collectively dampening immune responses through multiple pathways [28].

#### Novel immune checkpoints

In recent years, researchers have identified a series of novel immune checkpoint molecules that play crucial roles in modulating T cell function and tumor immune evasion. These emerging checkpoints contribute to the immunosuppressive TME through distinct mechanisms, including metabolic regulation, induction of cellular exhaustion, and modulation of immune cell function.

1. Lymphocyte-activation gene 3 (LAG-3)
  LAG-3 is highly expressed in tumor-infiltrating lymphocytes of HCC patients, suggesting that it is a potential target for HCC immunotherapy [29]. LAG-3 not only negatively regulates T cell function but also enhances the immunosuppressive capacity of regulatory T cells (Tregs) [30]. It binds to MHC-II molecules, mediating trans-endocytosis to reduce the expression of MHC-II molecules on APCs. This process weakens antigen presentation and impairs CD4^+^ T cell activation [31]. Additionally, LAG-3 is predominantly expressed on exhausted CD8^+^ T cells in the TME, directly inhibiting their function [32].
2. T cell immunoglobulin and mucin-domain containing-3 (TIM-3)
  TIM-3 is highly expressed in HCC tissues, where it binds to galectin-9 (Gal-9) to suppress CD8^+^ T cell function and reduce IFN-γ secretion [33]. TIM-3 also promotes the polarization of macrophages toward the immunosuppressive M2 phenotype, further dampening the anti-tumor immune response [34]. In Tregs, the interaction between Galectin-9 and TIM-3 on Th1 cells suppresses Th1 cell function [35].
3. B and T lymphocyte attenuator (BTLA)
  Increased BTLA expression on cytotoxic CD8^+^ T cells is associated with high postoperative recurrence rates in HCC [36]. The primary ligand of BTLA is the herpesvirus entry mediator (HVEM). Their interaction recruits SHP-1 phosphatase, which mediates immune suppression by dephosphorylating intracellular signaling proteins [37]. For instance, when BTLA interacts with the B cell receptor (BCR), it recruits SHP-1 via HVEM, thereby decreasing the activation of downstream BCR signaling molecules and raising the activation threshold for B cells [38]. Furthermore, the BTLA-HVEM interaction suppresses CD4^+^ T cell activation, proliferation, and cytokine production by activating the phosphoinositide 3-kinase/protein kinase B (PI3K/AKT) pathway, ultimately promoting apoptosis [37].
4. V-domain Ig suppressor of T cell activation (VISTA)
  VISTA selectively binds to P-selectin glycoprotein ligand-1 (PSGL-1) and inhibits T cell function under acidic pH conditions [39]. As a direct target of the SP1-YAP/TEAD4 complex, VISTA is widely expressed in cancer cells. Its upregulation promotes an immunosuppressive TME by inhibiting CD8^+^ T cell anti-tumor activity, thereby facilitating YAP/TAZ-driven immune evasion [40]. Additionally, VISTA suppresses TLR-mediated immune responses by inhibiting MAPK/AP-1 and IKK/NF-κB signaling cascades, leading to reduced cytokine release [41]. VISTA also enhances the immunosuppressive function of myeloid-derived suppressor cells (MDSCs) through STAT3 and polyamine-dependent mechanisms, playing a pivotal role in HCC immune evasion [42].
5. T cell immunoreceptor with Ig and ITIM domains (TIGIT)
  TIGIT interacts with its ligands CD155 and CD112 to exert its immunosuppressive effects. In DCs, TIGIT induces CD155 phosphorylation, triggering a signaling cascade that reduces IL-12 production while increasing IL-10 secretion, thus promoting the formation of tolerogenic DCs [43]. In tumor-infiltrating NK cells, TIGIT is highly expressed and its balance with DNAM-1 regulates NK cell cytotoxicity via PI3K-AKT-ERK phosphorylation cascades, thereby shaping the immune response [44]. Additionally, TIGIT expression on Tregs enhances cancer metastasis and suppresses anti-tumor immunity by inducing IL-32 expression [45].
6. Natural killer group 2A (NKG2A)
  NKG2A is primarily expressed on NK cells and interacts with HLA-E to maintain NK cell inhibition, preventing cytotoxic activity against target cells [46, 47] (Fig. 1A). IL-10 specifically induces NKG2A expression in NK cells, promoting their exhaustion, which correlates with poor prognosis in HCC patients [47, 48]. In the TME, tumor-specific T cell activation induces NKG2A expression, which interacts with HLA-E to transmit inhibitory signals, facilitating immune evasion [49].
7. Inositol 1,4,5-trisphosphate receptor interacting protein like 1 (ITPRIPL1)
  Recently, a new immune checkpoint, ITPRIPL1, has been found to bind to and interact with the CD3ε subunit on the surface of T cells, reducing the inflow of calcium into the T cell membrane and weakening the T cell response [50]. The mRNA expression levels of ITPRIPL1 progressively increase across different pathological stages of liver hepatocellular carcinoma (LIHC), suggesting its potential role in tumor progression [51].
8. Immunoglobulin superfamily member 8 (IGSF8)
  IGSF8 has been found to inhibit the killing of tumor cells by NK cells by binding to the MHC-I receptor KIR3DL2 on NK cells [52]. The IGSF8 inhibitor GV20-0251 has entered clinical trials and has demonstrated safety, tolerability, and preliminary clinical efficacy in patients with heavily pretreated advanced solid tumors [53].
9. Odd-skipped related 2 (Osr2)
  Osr2 functions as a key immune checkpoint in CD8^+^ T cells in response to mechanical stress by being upregulated via the mechanosensitive ion channel protein Piezo1. Osr2 recruits histone deacetylase 3 (HDAC3) to reconfigure the epigenetic program, thereby suppressing cytotoxic gene expression and promoting CD8^+^ T cell exhaustion [54]. The transcriptional and epigenetic reprogramming mediated by Osr2 may act in concert with the NFAT/TOX signaling pathway, contributing to T cell exhaustion and dysfunction within tumors [55]. However, its application in HCC remains in an immature stage.
  These immune checkpoint molecules collectively contribute to the fine-tuning of immune responses and represent potential targets for therapeutic interventions in various immune-related disorders, including cancer immunotherapy.

### Role of immune checkpoint in TME

Immune checkpoints are closely related to the TME, and the development of cancer is a subtly coordinated interaction with the TME (Fig. 1B). The TME is infiltrated by a myriad of innate and adaptive immune cells, which can be categorized into antitumor effector cells and immunosuppressive cells [56]. The balance between these immune cell subsets directly impacts tumor growth, dissemination, and response to therapies [57]. Tumor cells employ various strategies to dampen immune cell attacks, including downregulating the activity of stimulatory immune receptors and upregulating the activity of inhibitory immune receptors [58]. For instance, tumor cells can diminish surface MHC-I levels to downregulate signaling through TCR and can also upregulate surface PD-L1 levels to modulate signaling via PD-1, thereby exerting inhibitory effects [13, 59]. Disrupting the activation of inhibitory immune receptors can reactivate immune cells’ antitumor functions [59, 60]. Both liver cancer cells and immune cells express immunosuppressive molecules, whose interactions serve to suppress antitumor immune responses [61]. Furthermore, liver cancer tissues harbor abundant immunosuppressive cells such as Tregs, MDSCs, and M2-type macrophages. These cells inhibit the activity of antitumor effector cells, thus attenuating immune cells’ ability to attack tumors.

1. Tregs
  Tregs exhibit immunosuppressive functions and are highly enriched in the TME, where they express elevated levels of immune checkpoint molecules such as PD-L1 and CTLA-4. Their accumulation contributes to the suppression of T cell activity [62]. PD-1/PD-L1 signaling promotes lipid metabolism, proliferation, and suppression pathways in tumor-infiltrating Tregs. Furthermore, Tregs expressing CTLA-4 are more likely to engage with co-stimulatory molecules (CD80 and CD86) on APCs, thereby depriving T cells of stimulatory signals. Moreover, Tregs can induce DC apoptosis, further impairing the immune response [63]. Therefore, immune checkpoint blockade or inhibition can enhance antitumor immunity by weakening the stability and metabolic adaptability of Tregs lineages in the TME [64].
2. NK cells
  In HCC patients, PD-1 is upregulated in tumor-infiltrating NK cells and is associated with poor prognosis. By interacting with PD-L1 on tumor cells, PD-1 induces NK cell apoptosis and disrupts the PI3K/AKT signaling pathway, leading to NK cell inhibition [65, 66]. More immediately, blocking PD-1/PD-L1 enhances the immune function of NK cells [67].
3. MDSCs
  The MDSCs represent a heterogeneous population of bone marrow-derived cells capable of inhibiting antitumor immune responses mediated by T cells and NK cells while promoting tumor growth [68]. On one hand, MDSCs upregulate the metabolic activities of inducible nitric oxide synthase (iNOS) and arginase-1 (Arg-1), leading to L-arginine depletion, which inhibits T cell proliferation. Additionally, MDSCs generate reactive oxygen species (ROS) to suppress T cell immune responses [69]. On the other hand, MDSCs recruit Tregs and interact with the inhibitory receptor PD-1 to block T cell and NK cell activity [70]. Furthermore, MDSCs suppress T cells via TIM-3 and TIGIT signaling while secreting IL-10, which induces PD-L1 upregulation in Tregs, thereby reinforcing immunosuppressive effects [44].
4. M2 tumor-associated macrophages (TAMs)
  Macrophages are essential cells in the TME in liver cancer, which can be polarized to disparate functional phenotypes, such as M1 and M2. In general, it is believed that M2 TAM plays a pro-tumor role while M1 TAM exhibits antitumor function [71]. TAM polarizes into M2 by secreting TGF-β and upregulates the expression of PD-1 and PD-L1, depleting T lymphocytes [72] (Fig. 1B).
5. Regulation by cytokines and soluble factors
  The local environment of cytokines and other soluble mediators also determines the composition and function of HCC immune infiltration. Secretion of soluble immunosuppressive factors such as TGF-β, IL-10, reactive oxygen species (ROS) and nitric oxide (NO) by malignant cells themselves as well as immune cells in the TME promotes immunosuppression [10, 28].
  IL-10, produced by DCs, TAMs, Tregs, and B cells, inhibits the antigen-presenting ability of APCs and modulates cell proliferation. Met is a tyrosine kinase that plays a crucial role in the connection between IL-10 and PD-L1. When IL-10 activates the Met pathway, it can upregulate PD-L1 expression [25]. Moreover, IL-10 also promotes upregulation of PD-L1 in monocytes, and impairs the recruitment of tumour-specific T cells [73]. IFN-γ is key to an effective antitumour immune responses and is primarily produced by CD4^+^ T cells, cytotoxic CD8^+^ T cells, NK cells, and NKT cells. It induces tumor cell apoptosis via IRF1 and promotes non-apoptotic cell death in HCC through autophagy induction [74]. However, prolonged IFN-γ exposure can paradoxically promote malignant transformation by inducing PD-L1 and PD-L2 expression, leading to immune evasion. Additionally, IFN-γ has been shown to strongly upregulate PD-L1 expression on ovarian tumor cells, resulting in T cell inactivation and further exacerbating tumor immune evasion [75]. In patients with HCC, lower serum levels of IFN-γ are associated with more advanced tumour stage and worse prognosis [73] (Fig. 1B).
  As mentioned above, immune checkpoints are deeply involved in almost all aspects of immune responses within the TME. The role of immune checkpoints in immune responses is so extensive and critical that there is no doubt that intervening with immune checkpoints holds promising prospects as an anti-tumor therapeutic strategy.

## Clinical application of ICIs in HCC

ICIs are monoclonal antibodies that block the interaction of checkpoint proteins with their ligands, thereby preventing the inactivation of T cells [73]. HCC is prone to metastasis, recurrence, and drug resistance, which are the main obstacles in clinical treatment [76]. ICIs have demonstrated unprecedented therapeutic efficacy in clinical trials, offering breakthroughs in cancer treatment. Among these, CTLA-4 inhibition and PD-1/PD-L1 blockade are the most commonly employed checkpoint blockade strategies. CTLA-4 primarily regulates T cell activation by competing with CD28 for ligand binding during the early priming phase, whereas PD-1 mainly functions in peripheral tissues by limiting T cell activity and promoting T cell exhaustion in advanced HCC. The treatment strategies of ICIs can be simply divided into monotherapy with single ICIs and combination therapy with multiple ICIs. The following sections will elaborate on each approach.

### Monotherapy with single ICIs

#### PD-1/PD-L1 inhibitors

The development of PD-1/PD-L1 inhibitors has significantly advanced immunotherapy for HCC, offering new treatment options beyond traditional tyrosine kinase inhibitors (TKIs). PD-1/PD-L1 inhibitors Nivolumab, Pembrolizumab, Sintilimab, Camrelizumab, Atezolizumab and Durvalumab have received FDA approvals since 2011 [77]. Clinical trials evaluating these agents have provided valuable insights into their efficacy and limitations in both first- and second-line treatment settings.

Among these inhibitors, Nivolumab has demonstrated promising clinical activity, particularly in patients with advanced HCC. A open-label, Phase III trial, CheckMate 459, revealed a trend for a longer overall survival (OS) with Nivolumab versus Sorafenib in the first line (16.4 vs 14.7 months; hazard ratio (HR) 0.85; 95% CI 0.72–1.02). However, the difference did not reach statistical significance (*P* = 0.075) [78]. Nevertheless, Nivolumab demonstrated clinical activity and a favorable safety profile in patients with advanced HCC [79]. Furthermore, five-year follow-up data from the CheckMate 040 study confirmed that Nivolumab monotherapy provided sustained clinical benefits with manageable safety, regardless of prior Sorafenib treatment [80]. Based on the data from CheckMate 040 and CheckMate 459, Nivolumab shows superior OS compared to Sorafenib, with a median OS of 16.4 months. Notably, in CheckMate 459, Nivolumab achieved a median OS of 26.6 months in Sorafenib-naive patients, highlighting its potential in both first- and second-line settings, particularly for patients who are unsuitable for or intolerant to Sorafenib.

Similarly, Pembrolizumab has shown efficacy in the second-line setting. In the KEYNOTE-224 trial, Pembrolizumab achieved a median OS of 12.9 months and an ORR of 16.3% [81]. Further evidence from KEYNOTE-240 demonstrated that Pembrolizumab extended median OS to 13.9 months, outperforming the placebo group (10.6 months; HR 0.78; *P* = 0.018) [82]. Moreover, the KEYNOTE-394 trial showed that Pembrolizumab achieved a median OS of 14.6 months, significantly better than the placebo group (13.0 months; HR 0.79; *P* = 0.0180), with a notably higher ORR (12.7% vs 1.3%) [83]. These data indicate that Pembrolizumab can significantly prolong survival and improve ORR in the second-line setting, with a manageable safety profile. However, other PD-1/PD-L1 inhibitors have shown limited efficacy as monotherapies. For instance, Tislelizumab (RATIONALE-208 trial) and Sintilimab (ChiCTR2000037655 trial) exhibited some clinical activity, but both had ORRs below 20% and relatively short PFS.

While monotherapy with PD-1/PD-L1 inhibitors has demonstrated clinical benefits in HCC, particularly in the second-line setting, its efficacy remains constrained by low ORR and short PFS. This underscores the need for combination strategies to enhance treatment outcomes. Among the approved agents, Nivolumab has shown the most promising results in HCC, with durable responses and notable survival benefits, particularly in Sorafenib-naïve patients. Given the limitations of monotherapy, ongoing research is focused on optimizing combination approaches to further improve therapeutic efficacy in HCC.

#### CTLA-4 inhibitors

Two CTLA-4 specific fully human monoclonal antibodies, namely Tremelimumab and Ipilimumab, have so far reached the clinical phase of experimentation for the treatment of human cancer [14]. Ipilimumab was the first FDA-approved ICIs in 2011 for patients suffering from advanced melanoma [84]. Ipilimumab avoids T cell suppression and stimulates the effector T cell activation and proliferation. This antibody, in combination with Nivolumab as the PD-1 inhibitor, has been approved for the treatment of patients with metastatic colorectal cancer (CRC) [85]. In the CheckMate 040, combining with Ipilimumab may improve clinical outcomes compared with Nivolumab monotherapy. The arm (4 doses Nivolumab 1 mg/kg plus Ipilimumab 3 mg/kg every 3 weeks then Nivolumab 240 mg every 2 weeks) received accelerated approval in the US based on the results of this study [86]. In 2015, Tremelimumab was granted Fast Track Designation and Orphan Drug Designation by the FDA as a potential treatment for malignant mesothelioma [87]. Furthermore, Tremelimumab has shown significant antitumor activity and overall safety in patients with advanced melanoma and is widely used in early clinical studies of gastric, pancreatic, breast, and non-small cell lung cancers [88–92]. Notably, Tremelimumab is often used as a CTLA-4 inhibitor in combination with other drugs to treat HCC [93]. In a population of patients with HCC superimposed on chronic HCV infection with fairly advanced tumors, Tremelimumab showed a safety profile and signs of antitumoral and antiviral effects (ORR 17.6%, TTP 6.5 months, mOS 8.2 months; 95% CI 4.64–21.34) [94]. These two CTLA-4-specific antibodies have demonstrated robust antitumor activity and a favorable safety profile in the treatment of various cancers, and their combination with other therapies may further enhance clinical outcomes.

CTLA-4 inhibitors, such as Ipilimumab and Tremelimumab, are primarily used in combination therapy, significantly enhancing ORR and OS when paired with PD-1 inhibitors. While PD-L1 inhibitors dominate HCC immunotherapy, CTLA-4 inhibitors demonstrate robust antitumor activity, particularly in advanced HCC and other cancers, offering a promising approach for improving clinical outcomes through synergistic immune modulation.

Monotherapy with immune checkpoint inhibitors has made significant progress in cancer treatment, but it also exhibits certain limitations. As shown in Table 1, the ORR of single-agent immune checkpoint inhibitor therapy does not exceed 20%. Not all patients achieve durable treatment responses, and some Phase III trials have failed to meet their primary endpoints. For example, the CheckMate 459 trial did not achieve its OS primary endpoint, possibly due to tumor heterogeneity in HCC, which may have led to suboptimal responses to Nivolumab in some patients [95]. Additionally, the lack of statistical significance in the benefit of Nivolumab over Sorafenib in specific patient subgroups may be attributed to trial design, patient selection criteria, and the immunosuppressive state of the TME. Furthermore, compared to other Phase III studies, such as IMbrave150, the CheckMate 459 trial did not demonstrate a clear survival advantage, highlighting the limitations of single-agent PD-1 inhibitors in first-line HCC treatment. Moreover, the PFS in most studies ranged only between 2 and 4 months, indicating limited efficacy in controlling disease progression. Compared to CTLA-4 inhibitors, PD-1/PD-L1 inhibitors are more widely used in HCC, and although they are generally well-tolerated, they may still cause immune-related adverse events (irAEs), such as fatigue, rash, gastrointestinal reactions, and autoimmune thyroid diseases. In severe cases, immune-related inflammation may occur [96, 97]. To overcome these challenges, future research should focus on refining patient selection criteria and exploring combination therapy strategies to enhance the efficacy and safety of ICIs in HCC.

**Table 1:** Outcomes of ICI therapy for HCC

Clinical trial information		Treatments (trial)	Phase	n	ORR	mPFS (months)	mOS (months)	Treatment-related adverse event rates		Median follow-up (months)
								Grade ≥ 3	Most common grade 3–4	
Single ICI therapy	NCT01658878 (CheckMate 040) [80]	Nivolumab (Sorafenib-naive)	I/II	80	20%	22.6 (DOR)	26.6	33%	Elevated serum AST (10%) and/or ALT (6%)	60
		Nivolumab (Sorafenib-experienced)	I/II	154	14%	39.7 (DOR)	15.1	21%	Elevated serum AST (4%) and/or ALT (3%)	60
	NCT02576509 (CheckMate 459) [78]	Nivolumab vs Sorafenib	III	371 vs 372	15% vs 7%	3.8 vs 3.9	16.4 vs 14.7	12% vs 11%	Hypertension (0 vs 7%) and elevated serum AST (6% vs 4%)	15.2 vs 15.2
	NCT02702414 (KEYNOTE-224) [81]	Pembrolizumab	II	104	16.3%	4.8	12.9	25%	Fatigue (21.2%) and increased AST (12.5%)	8.4
	NCT02702401 (KEYNOTE-240) [82]	Pembrolizumab vs placebo	III	278 vs 135	18.3% vs 4.4%	3.3 vs 2.8	13.9 vs 10.6	52% vs 46.3%	No new or unexpected AEs occurred	39.6 vs 39.8
	NCT03062358 (KEYNOTE-394) [213]	Pembrolizumab vs placebo	III	300 vs 153	12.7% vs 1.3%	2.6 vs 2.3	14.6 vs 13.0	66.9% vs 49.7%	Elevated serum AST (2.3% vs 2.0%) and/or ALT (1.7% vs 1.3%)	33.8 vs 33.8
	NCT01008358 [94]	Tremelimumab	II	21	17.6%	6.5 (TTP)	8.2	45%	Elevated serum AST (45%) and/or ALT (25%); hyponatraemia (30%)	NR
	ChiCTR2000037655 [174]	Sintilimab vs active surveillance	II	99 vs 99	NR	27.7 vs 15.5 (RFS)	NR	12.4%	Elevated ALT (5.2%) and anemia (4.1%)	23.3 vs 23.3
	NCT03419897 (RATIONALE-208) [214]	Tislelizumab	NR	235	13.6%	2.7	13.5	49.4%	Elevated ALT (28.1%) and anemia (20.9%)	12.5
	ChiCTR2000041405 [215]	Camrelizumab	A real-world study	41	29.3%	NE, expected more than 9 months	NR	51.2%	Increased gamma-glutamyltransferase (36.6%) and increased AST (17%)	5.28
Dual ICI therapy	NCT01658878 [86]	Nivolumab + Ipilimumab	I/II	50	32%	17.5 (DOR)	22.2	53%	Elevated serum AST (16%); lipase (12%) and/or ALT (8%)	30.7
	NCT02519348 [105]	Tremelimumab + Durvalumab	I/II	75	24.0%	2.17	18.73	37.8%	Elevated serum AST (16%); lipase (12.2%) and/or ALT (4.1%)	NR
	NCT03298451 [216]	Tremelimumab + Durvalumab	III	393	20.1%	3.8	16.4	25.8%	NR	16.1
	NCT03222076 [102]	Nivolumab + Ipilimumab vs Nivolumab	II	14 vs 13	NR	19.53 vs 9.4	NA	43% vs 23%	Elevated serum AST (50% vs 23%) and/or ALT (50% vs 23%)	24.6 vs 24.6
	NCT04039607 (CheckMate 9DW) [103]	Nivolumab + Ipilimumab vs Lenvatinib or Sorafenib	III	335 vs 333	36% vs 13%	30.4 vs 12.9 (DOR)	23.7 vs 20.6	41% vs 42%	NR	35.2 vs 35.2

*ALT* alanine aminotransferase, *AST* aspartate aminotransferase, *DOR* duration of response, *mOS* median overall survival, *mPFS* median progression-free survival, *n* number of patients, *NA* not available, *NE* not evaluable, *NR* not reported, *ORR* objective response rate, *RFS* recurrence-free survival, *TTP* time to progression

Identifying patients most likely to benefit from monotherapy remains crucial for optimizing treatment outcomes. Patients with well-preserved liver function (Child–Pugh class A) or those with a high infiltration of T cells and PD-L1 expression in the TME are more likely to benefit from monotherapy [98]. For patients who maintain a good performance status (ECOG PS 0–1) after first-line TKI failure and are unsuitable for combination therapy, PD-1/PD-L1 monotherapy, such as Pembrolizumab or Nivolumab, may be considered as a second-line option [81]. While ICIs have revolutionized HCC treatment, optimizing their use through patient stratification and combination approaches remains essential for maximizing clinical benefit.

### Polytherapy with multiple ICIs

Although monotherapy with ICIs has demonstrated certain efficacy in the treatment of hepatocellular carcinoma HCC, the immunosuppressive nature of the TME and the development of resistance mechanisms often limit the durability of antitumor responses [99]. Consequently, combination therapy strategies have emerged as a more promising approach, particularly the dual inhibition of CTLA-4 and PD-1/PD-L1, which has shown superior efficacy and prolonged survival benefits in multiple clinical studies. This success may be attributed to the distinct roles of CTLA-4 and PD-1 in different stages of T cell immunity, where they regulate T cell activity in different manners. CTLA-4 is considered the “leader” among immune checkpoint inhibitors, as CTLA-4 blockade primarily regulates T cell activation in the early stages within lymphoid tissues and suppresses DCs activity through Tregs [100]. In contrast, PD-1 inhibitors function later in the immune response by modulating T cell activity in peripheral tissues through interactions with PD-L1 and PD-L2 and by promoting Treg differentiation [101]. Consequently, the simultaneous blockade of CTLA-4 and PD-1 represents a rational and synergistic combination therapy, capitalizing on their unique mechanisms of action [87].

In the field of HCC treatment, the combined use of immune checkpoint inhibitors is becoming increasingly important. In a multicenter, open-label, multicohort Phase I/II study known as CheckMate 040, the combination of Nivolumab and Ipilimumab achieved an ORR of 32.0% in patients with unresectable HCC [86]. Kaseb et al. randomized resectable HCC patients (1:1) to receive either intravenous Nivolumab alone or Nivolumab at 240 mg every 2 weeks plus a single dose of Ipilimumab at 1 mg/kg. Both the monotherapy and combination therapy with Nivolumab were deemed safe and feasible. The median PFS was 9.4 months (95% CI: 1.47-NE) in the Nivolumab group and 19.53 months (95% CI: 2.33-NE) in the combination group (HR 0.89; 95% CI: 0.31–2.54) [102]. Given these findings, the combination of Nivolumab and Ipilimumab has received accelerated approval from the FDA as a second-line treatment following Sorafenib. Moreover, the Phase III CheckMate 9DW trial further evaluated the efficacy of this combination in patients with unresectable HCC. The results demonstrated that first-line treatment with Nivolumab plus Ipilimumab led to a statistically and clinically significant improvement in median OS compared to Lenvatinib or Sorafenib (23.7 vs 20.6 months; HR 0.79; 95% CI: 0.65–0.96; *P* = 0.0180). Additionally, the combination therapy group exhibited a markedly higher ORR (36% vs 13%), highlighting its potential as a novel first-line treatment option for advanced HCC [103].

In December 2022, Tremelimumab in combination with Durvalumab received a positive opinion from the European Union Committee for Medicinal Products for Human Use as a first-line treatment for adults with advanced or unresectable HCC [14, 104]. For example, a Phase I/II study evaluated the efficacy of Tremelimumab and Durvalumab as monotherapy and combination therapy in unresectable HCC patients, with the primary endpoint being safety. The combination therapy group exhibited superior efficacy, with an ORR of 24%, a median DOR not reached, and a median OS of 18.73 months [105]. Besides, In the HIMALAYA trial, combination therapy consisting of Durvalumab and Tremelimumab also exhibited improved OS rates (16.4 vs 13.8 months, HR 0.78) in comparison to Sorafenib [106]. The results of the Phase III HIMALAYA and IMbrave150 trials suggest that Atezolizumab/Bevacizumab is generally the preferred first-line treatment. However, in cases where disease progression occurs during Atezolizumab/Bevacizumab therapy, the combination of Durvalumab and Tremelimumab can be considered as second-line therapy [106]. Notably, Durvalumab and Tremelimumab represent the first anti-PD-L1 plus anti-CTLA-4 combination immunotherapy regimen to demonstrate success in a Phase III clinical trial. In summary, the combination therapy of immune checkpoint inhibitors has shown promise in the treatment of HCC.

In the selection of immunotherapy regimens, the aforementioned clinical trial data provide crucial evidence regarding the efficacy and safety of different treatment strategies. The IMbrave150 trial demonstrated that Atezolizumab in combination with Bevacizumab exhibits significant therapeutic efficacy in the first-line treatment of advanced HCC. For second-line therapy, data from the CheckMate 459 and CheckMate 040 trials indicate that Nivolumab monotherapy demonstrates substantial clinical activity and a favorable safety profile in the treatment of advanced HCC. Moreover, the combination of Nivolumab and Ipilimumab has shown superior efficacy in patients who experience disease progression, achieving an ORR of 32%. Further evidence from the CheckMate 040 and CheckMate 9DW trials suggests that Nivolumab plus Ipilimumab outperforms Nivolumab monotherapy in terms of both ORR and OS. Similarly, findings from the HIMALAYA trial and Phase I/II studies indicate that the combination of Tremelimumab and Durvalumab offers promising clinical benefits for patients who progress after treatment with Atezolizumab plus Bevacizumab. For patients who are unable to tolerate dual immunotherapy, data from the KEYNOTE-224 and KEYNOTE-240 trials further support the role of Pembrolizumab monotherapy in the second-line setting, demonstrating superior antitumor activity and survival benefits compared to placebo.

Despite the reported efficacy of ICIs, there are still potential serious adverse effects associated with their use (Table 1) [102, 105–107]. Apart from common side effects like fever, cough, rash, and fatigue, additional symptoms may arise, necessitating further optimization of treatment strategies based on patient-specific clinical data indicators. There are multiple reasons why ICI therapy can lead to severe adverse reactions. Firstly, these drugs enhance the immune system’s ability to attack tumors by activating it, however, this also increases the risk of the immune system attacking normal tissues. Secondly, the disruption of immune system balance by ICIs may result in excessive immune activation, triggering irAEs such as immune-mediated inflammation and autoimmune diseases. Additionally, individual variations in immune system status can influence how a person responds to ICIs, thereby escalating the risk of adverse reactions. Consequently, close monitoring of irAEs in patients is essential when utilizing immune checkpoint inhibitors, allowing for tailored adjustments and management based on patient status.

When selecting an immunotherapy regimen, clinicians must consider several factors, including tumor burden, liver function status (such as Child–Pugh score), prior treatment history, and relevant biomarkers. In the first-line setting, combination immunotherapy is generally preferred, as exemplified by the IMbrave150 trial, which demonstrated the efficacy of Atezolizumab plus Bevacizumab. In the second-line setting, for patients who experience disease progression after first-line immunotherapy, the combination of Nivolumab and Ipilimumab has shown promising efficacy, with an ORR of 32%, as reported in the CheckMate 040 trial. Alternatively, the HIMALAYA trial has provided evidence supporting the use of Tremelimumab plus Durvalumab as a second-line option for patients whose disease progresses following treatment with Atezolizumab and Bevacizumab. For patients who are unable to tolerate dual immunotherapy, monotherapy with immune checkpoint inhibitors, such as Pembrolizumab, may be considered. Data from the KEYNOTE-240 trial further support its role as a viable second-line treatment option.

### Mechanisms of resistance to ICIs in HCC

Despite the significant ORR observed with ICIs in various cancers, many patients with HCC exhibit primary resistance or develop acquired resistance following an initial response. This phenomenon of immune resistance involves multiple complex mechanisms, including impaired antigen recognition and presentation by immune cells, abnormal activation and proliferation of immunosuppressive cells, elevated levels of inhibitory cytokines and chemokines, and dysfunction of antitumor immune cells within the TME. Additionally, loss of tumor antigen expression, tumor heterogeneity, and gut microbiota dysbiosis have been identified as factors closely associated with ICI resistance.

#### Aberrant expression and dysfunction of immune checkpoint molecules in HCC

The dysregulation of immune checkpoint molecules plays a significant role in both primary and acquired resistance to ICIs in HCC. These molecules regulate immune activation and tolerance, and their abnormal expression can lead to immune evasion by tumors. Understanding how immune checkpoints interact and influence ICI efficacy is crucial for optimizing therapeutic strategies.

PD-L1 expression has been widely used as a predictive biomarker for ICI efficacy in various cancers; however, its predictive value in HCC remains controversial. Studies have shown that PD-L1 interacts in cis with CD80, a process that not only blocks PD-L1/PD-1 signaling but also disrupts CD80 homodimerization, thereby reducing the affinity of the CD80/CTLA-4 interaction. When PD-L1 expression exceeds that of CD80, the PD-L1/CD80 cis-complex protects CD80 from trans-endocytosis, thereby attenuating CTLA-4 and Tregs functions, whereas unbound PD-L1 continues to activate the PD-1 signaling pathway. Thus, PD-L1 expression alone is insufficient to accurately predict HCC responsiveness to ICIs, and the relative expression levels of PD-L1 and CD80 may serve as better biomarkers for anti-CTLA-4 and anti-PD-1/PD-L1 therapies [108].

Another crucial mechanism underlying acquired resistance involves the compensatory upregulation of alternative immune checkpoint molecules. In HCC, upregulation of PVRL1 stabilizes the surface expression of PVR, which interacts with the inhibitory receptor TIGIT on CD8^+^ effector memory T cells, suppressing antitumor immune responses and leading to resistance to PD-1 inhibitors [109]. Furthermore, upregulation of inhibitory receptors such as TIM-3 and LAG-3 has also been implicated in the development of ICI resistance [13].

Aberrant immune checkpoint regulation, including PD-L1 expression dynamics and the upregulation of inhibitory receptors like TIGIT, TIM-3, and LAG-3, significantly contributes to ICI resistance in HCC. A deeper understanding of these molecular interactions could help refine patient selection and improve immunotherapy outcomes.

#### Regulation of the TME in HCC

The TME plays a pivotal role in determining the success or failure of ICIs in HCC. A highly immunosuppressive TME can hinder the activation and function of antitumor immune cells, leading to both primary and acquired resistance. Various cellular, molecular, and metabolic factors within the TME contribute to immune evasion and diminished ICI efficacy.

As previously mentioned, TAMs, Tregs, and MDSCs contribute to the formation of an immunosuppressive TME through various mechanisms. Research has demonstrated that anti-PD-1 therapy activates the PD-L1/NOD-like receptor family pyrin domain containing 3 (NLRP3) inflammasome signaling pathway in CD8^+^ T cells. This activation promotes the recruitment of polymorphonuclear MDSCs into tumor tissues, subsequently suppressing antitumor immune responses and leading to treatment resistance [110, 111].

Additionally, several molecular and metabolic factors within the TME contribute to primary resistance in HCC [12]. TGF-β, produced by tumor cells, macrophages, or Tregs, is associated with immunosuppression, and its expression in tissues is often indicative of poor prognosis in HCC patients [112]. TGF-β promotes IL-10 secretion, induces Tregs differentiation, and inhibits T cell function, while also polarizing TAMs toward an M2-like phenotype, thereby attenuating the efficacy of PD-1 inhibitors [13]. Moreover, aberrant activation of the WNT/β-catenin signaling pathway, which is widespread in HCC, reduces ICI efficacy by inhibiting DC recruitment, decreasing T cell infiltration, and suppressing NK cell activity [113]. Clinical studies have confirmed that HCC patients harboring WNT/β-catenin mutations do not respond to ICI therapy, providing direct evidence for this resistance mechanism [114].

Given the profound influence of the TME on ICI resistance in HCC, targeting immunosuppressive pathways such as TGF-β and WNT/β-catenin represents a promising strategy to enhance the effectiveness of immunotherapy. A deeper understanding of these mechanisms may help identify novel therapeutic targets and improve patient outcomes.

#### Potential strategies to overcome ICI resistance

Given the multifaceted mechanisms driving resistance to ICIs in HCC, identifying effective strategies to restore treatment sensitivity is critical. Emerging research highlights the potential of gut microbiota modulation, targeting tumor mutational burden (TMB), and combination therapies as promising approaches to enhance immunotherapy efficacy and improve patient outcomes.

One of the most intriguing areas of research involves the role of the gut microbiota in shaping antitumor immunity [115]. Clinical studies have demonstrated that fecal microbiota transplantation can reverse resistance to PD-1 inhibitors in melanoma patients [116]. In HCC, the use of antibiotics during the early stages of ICI therapy has been associated with reduced treatment efficacy, suggesting that the gut microbiota plays a crucial role in modulating ICI response [117]. Certain gut microbiota species have been shown to enhance ICI efficacy in HCC by promoting the production of CXCL16 within the TME, thereby facilitating the recruitment of NKT cells [118]. *Bifidobacterium* has also been reported to enhance immune cell function and increase tumor infiltration, thereby improving the efficacy of anti-PD-1 therapy [119]. Furthermore, the gut microbiome may overcome resistance to PD-1 pathway inhibitors by downregulating PD-L2 and its binding partner, repulsive guidance molecule B (RGMb) [120].

In addition to microbiota modulation, TMB plays a key role in determining ICI sensitivity. Tumors with low TMB generate fewer neoantigens, leading to weak T cell responses and reduced immunogenicity, which contribute to ICI resistance [121]. Given that ICIs have been introduced relatively recently in HCC treatment, basic research and clinical trials investigating ICI resistance mechanisms remain limited, and available reference data are scarce. Although the precise mechanisms of resistance are not yet fully elucidated, combination therapies have shown promise in mitigating ICI resistance compared to monotherapy. For instance, chemotherapy and radiotherapy can enhance ICI efficacy by inducing tumor cell death, releasing neoantigens, and improving T cell priming, activation, and function [122]. Currently, combination therapy has become a primary treatment strategy for HCC, as it not only improves ORR but also effectively addresses the issue of ICI resistance.

Overcoming ICI resistance in HCC requires a multifaceted approach that includes gut microbiota modulation, targeting tumor mutational burden, and leveraging combination therapies. Continued research into these strategies will be essential for optimizing immunotherapy and improving clinical outcomes for patients with HCC.

## Clinical application of ICIs with conventional therapies in HCC

**Fig. 2:**
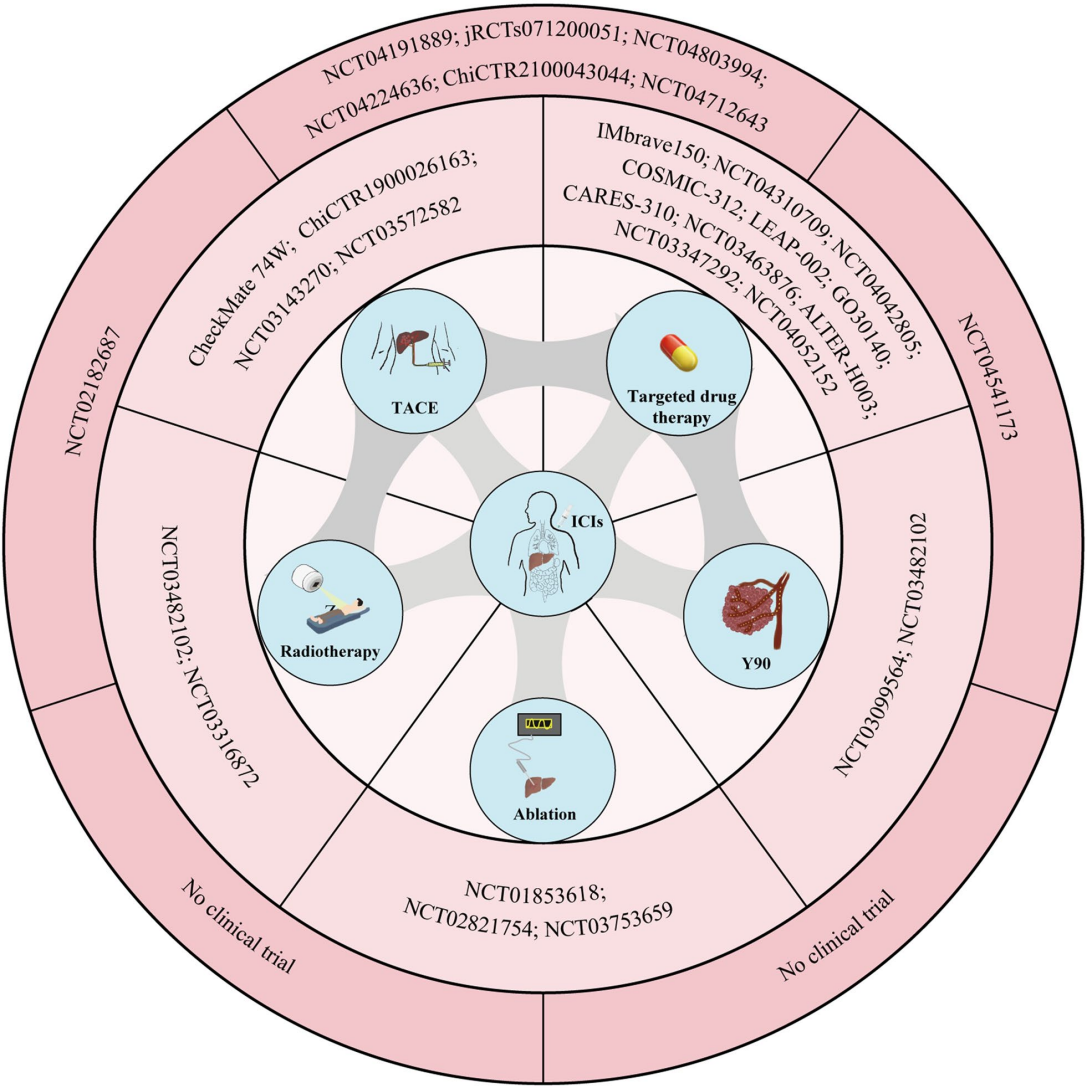
Combination therapy with ICIs. ICIs have shown great advantages in the immunotherapy of HCC. The combination of ICIs and other therapeutic methods has become a new trend in the treatment of HCC, and a number of clinical studies have proved that the combination of ICIs. Among these, clinical trials such as IMbrave150 and others, have investigated the combined use of ICIs and targeted drug therapies. Additionally, clinical trials involving the combined use of ICIs and Yttrium-90 (Y90), such as NCT03099564 and others, have shown promising results. Furthermore, clinical trials exploring the therapeutic potential of combining ICIs with ablation procedures are represented by trials such as NCT01853618 and others. Research into the combination of ICIs with radiotherapy is exemplified by trials including NCT03482102 and others. Moreover, investigations into the efficacy of combining ICIs with TACE are demonstrated by trials like CheckMate 74W and others. There are more and more clinical trials on ICIs combined with a variety of treatment modalities. There are clinical trials specifically examining the combination of ICIs with targeted drugs and TACE, as demonstrated by trials such as NCT04191889 and others. Similarly, ongoing clinical research includes investigations into the combination of ICIs with targeted drugs and Y90, as evidenced by trial NCT04541173. Furthermore, there are clinical trials exploring the combination of ICIs with radiotherapy and TACE, as illustrated by trial NCT02182687. These clinical trials collectively contribute to advancing our understanding of potential synergies of ICIs in the treatment of HCC

At present, the overall response rate of ICIs is not high, only 20 to 30% [123]. Furthermore, while ICIs enhance antitumor immune responses by relieving T cell suppression, this mechanism lacks tumor specificity. As a result, some T cells recognizing self-antigens may become activated, leading to irAEs. These immune-related toxicities can affect multiple organ systems, including the skin, gastrointestinal tract, liver, lungs, and endocrine system. In severe cases, irAEs may result in immune-related colitis, pneumonitis, hepatitis, and neurotoxicity, with potentially life-threatening consequences [124]. Moreover, not all HCC patients benefit from ICI therapy, which may be attributed to the immunosuppressive TME, tumor heterogeneity, and the lack of effective predictive biomarkers [87].Therefore, to overcome the limitations of ICI monotherapy, improve clinical efficacy, and mitigate treatment-related adverse events, combination strategies have emerged as a key focus in the development of HCC immunotherapy (Fig. 2). The fundamental rationale for ICI-based combination therapy lies in integrating different therapeutic mechanisms to counteract immune evasion, enhance antitumor immunity, and modulate the TME. Currently, the main combination strategies in HCC include: (1) ICIs combined with other immune checkpoint blockade, such as PD-1/PD-L1 inhibitors combined with CTLA-4 inhibitors, to enhance T cell activation and overcome tumor immune escape; (2) ICIs combined with locoregional therapies, which can promote antigen release and strengthen immune responses; (3) ICIs combined with targeted therapies, such as anti-angiogenic agents or TKIs, which help normalize tumor vasculature, increase T cell infiltration, improve the immune microenvironment, and overcome resistance. The exploration of these combination strategies not only has the potential to enhance the therapeutic efficacy of ICIs but may also reduce the incidence of immune-related adverse events, ultimately providing more optimized treatment options for patients with HCC.

### ICIs combined with interventional therapy

Interventional therapy, as a minimally invasive local treatment modality, encompasses transarterial chemoembolization (TACE), ablation, and radiotherapy, and has been widely utilized in the management of HCC [125]. While interventional therapy can induce local tumor necrosis and activate antitumor immune responses, it remains insufficient to completely prevent tumor recurrence or distant metastasis [126]. In recent years, the combination of interventional therapy with ICIs has brought new breakthroughs to HCC treatment. The synergistic mechanisms of this approach mainly include enhancing tumor antigen release to improve immunogenicity, modifying the TME, and boosting immune cell activity. Based on this, more interventional treatment methods have demonstrated significant value in the immunotherapy of HCC.

A study included 13 advanced HCC patients aimed to evaluate the short-term efficacy and safety of combined Atezolizumab plus Bevacizumab with interventional therapy as a first-line treatment for advanced HCC [127]. The study results showed an ORR of 30.8%, with a median PFS of 9.7 months (range 7.3–12.1 months), indicating potential antitumor effects of ICIs combined with interventional therapy. Simultaneously, a high level of effector memory CD8^+^ T cells may serve as a potential biomarker for predicting a favorable response to this combined treatment [128]. The effectiveness of interventional therapy varies depending on the tumor type. Studies have shown that local–regional interventional therapy can induce systemic immune responses but is not sufficient to effectively prevent local recurrence and distant metastasis.

#### ICIs combined with ablation techniques

Various types of ablation techniques, such as cryoablation and radiofrequency ablation (RFA), can be combined with ICIs to enhance antitumor immune responses [129] (Fig. 2). Cryoablation exerts its antitumor effects primarily through the stimulator of interferon genes (STING)-dependent type I interferon signaling pathway [130]. Moreover, this technique facilitates the uptake of tumor antigens by APCs, such as DCs, which subsequently migrate to lymph nodes and activate tumor-specific T cell responses. ICIs can further potentiate these responses by sustaining T cell activity, thereby improving therapeutic efficacy [131]. Notably, a study by Matthew T. et al. demonstrated that cryoablation combined with Tremelimumab significantly increased immune cell infiltration in clear cell renal cell carcinoma (ccRCC), suggesting that this combinatorial strategy is feasible and may induce favorable immunomodulatory changes within the TME [132].

On the other hand, RFA induces the release of large amounts of tumor-associated antigens, thereby promoting antitumor immune responses [133, 134]. Nevertheless, In the treatment of cholangiocarcinoma, the combination of ICIs with Durvalumab/Tremelimumab along with and without RFA showed good tolerability, as observed in the study by Cecilia Monge B. et al. However, the combination RFA group exhibited lower PFS and OS compared to the ICIs monotherapy group, with a PFS of 2 months (95% CI: 1.3–2.5) and 3.3 months (95% CI: 1.9–3.9), respectively, and an OS of 5.7 months (95% CI: 2.9–7.6) and 6.1 months (95% CI: 3.5–7.4) [135]. These findings suggest that the clinical efficacy of immune-based combination strategies may vary depending on tumor type and treatment protocol. Additionally, RFA has been shown to stimulate NK cell activation and differentiation, further augmenting the antitumor immune response [136]. Currently, a clinical trial is underway to explore the effects of RFA or TACE combined with ICI therapy on the immune system of patients with HCC. This study focuses on evaluating changes in immune parameters within tumor tissues and peripheral blood before and after combination therapy, with a particular emphasis on the role of NK cells in antitumor immunity [137]. The findings from this trial are expected to provide critical scientific insights for optimizing immune-based combination strategies.

#### ICIs combined with TACE

As an effective locoregional therapy, TACE has been widely utilized in the treatment of various solid tumors, and it remains the current standard of care for patients with intermediate-stage HCC. Tumor embolization during TACE induces necrosis of the tumor tissue, reducing the release of immunosuppressive factors and alleviating the inhibition of immune function. Additionally, embolization induces a hypoxic microenvironment, resulting in the upregulation of HIF-1α expression, increased expression of PD-L1 on the surface of immune cells and tumor cells [138]. The combination with ICIs can enhance therapeutic efficacy by restoring immune function. Additionally, TACE correlates with lower intratumoral exhausted CD8^+^PD-1^+^ effector cells and Tregs, and may transform an immunosuppressive microenvironment into an immunosupportive setting to enhance the response of PD-1/PD-L1 inhibitors [139]. Moreover, TACE facilitates the release of tumor antigens and induces microenvironmental changes that provide more favorable conditions for ICIs, ultimately enhancing their antitumor immune response.

A retrospective study evaluated the safety and efficacy of PD-1/PD-L1 inhibitors (Nivolumab) combined with TACE versus Nivolumab monotherapy. Compared to ICIs alone, the combination significantly prolonged PFS and OS, enhancing the response to PD-1/PD-L1 inhibitors. Specifically, the PFS of Nivolumab + TACE was 8.8 months, compared to 3.7 months for Nivolumab monotherapy (HR 0.47; 95% CI 0.27–0.84; log-rank *P* < 0.01), while OS was 35.1 months versus 16.6 months (HR 0.55; 95% CI 0.26–1.17; log-rank *P* = 0.12) [140]. Additionally, the study by Harding et al. confirmed the safety and antitumor activity of Nivolumab in combination with drug-eluting bead transarterial chemoembolization (DEB-TACE), with an ORR of 21%. Among the 19 patients studied, five patients experienced SD or better, with a duration of more than 12 months [141]. However, it is noteworthy that the combination therapy of ICIs with DEB-TACE may increase the risk of liver abscess formation if the interval is less than 30 days [142]. A clinical trial is evaluating whether TACE will enhance the clinical efficacy of Tremelimumab in the treatment of advanced HCC. Interim results showed that TACE combined with Tremelimumab is safe and feasible in the treatment of advanced HCC, and combination therapy could increase the infiltration of immune cells within the tumor site [143]. Jemma Buchalter et al. are also evaluating whether anti-CTLA4 doses on day 1 lead to a stronger immune response, so far they have found that Tremelimumab (Day 1 only) and Durvalumab in combination with TACE is safe and feasible in patients with HCC [144].

From the above clinical trials, it can be concluded that TACE enhances the antitumor effects of ICIs. A comparison of trials NCT03572582, ChiCTR1900026163, and NCT03143270 suggests that Nivolumab + TACE is the optimal combination, demonstrating the highest ORR (71.4%) and a relatively long PFS (7.2 months). Additionally, high levels of effector memory CD8^+^ T cells may serve as potential biomarkers for predicting favorable responses to this combined therapy [128].

In summary, the combination of interventional therapies (such as TACE and ablation) with ICIs has shown remarkable clinical potential in advanced HCC. Specifically, ablation techniques enhance antitumor immune responses by releasing tumor-associated antigens, thereby activating the immune system, whereas TACE induces tumor necrosis and modulates the TME, potentially augmenting the efficacy of ICIs. Clinical studies have indicated that TACE combined with ICIs can significantly prolong PFS and OS while improving patients' quality of life. However, the long-term efficacy and safety of these combination therapies require further validation, as their effectiveness may vary depending on tumor type and treatment regimen. Future research should focus on: (1) optimizing combination therapy strategies, including determining the optimal timing and dosage; (2) identifying predictive biomarkers, such as effector memory CD8^+^ T cells; and (3) conducting large-scale randomized controlled trials to further validate the long-term benefits of combination therapy. Through multidisciplinary collaboration and individualized precision therapy, further improvements in HCC patient outcomes are anticipated.

### ICIs combined with targeted drug therapy

**Fig. 3:**
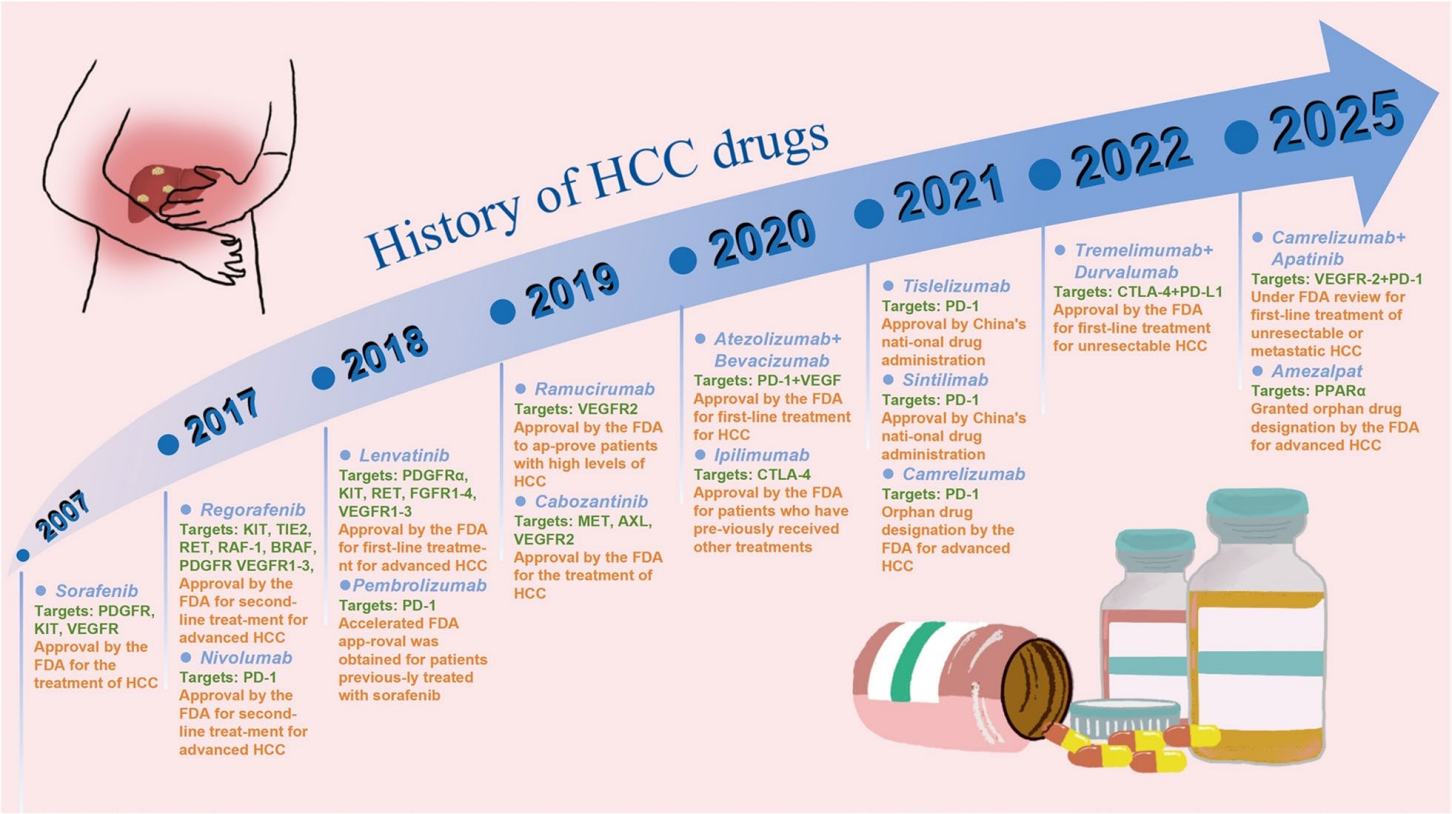
History of HCC drugs. Sorafenib is the first targeted therapy drug approved for the treatment of liver cancer. It was approved by the FDA in 2007 for the treatment of advanced HCC. From 2007 to 2017, there were no new breakthroughs in targeted therapy drugs for HCC. However, in recent years, a multitude of new drugs and innovative therapies have emerged, providing new options and hope for patients with advanced HCC

Targeted therapy represents a relatively novel approach in cancer treatment, focusing on specific molecular markers unique to tumor cells to inhibit their growth and dissemination. Figure 3 illustrates the historical development of HCC therapeutics. The combination of targeted therapy and immunotherapy enables a multifaceted attack on tumors. The synergistic effects of ICIs and targeted agents primarily manifest in the following aspects: First, targeted drugs can normalize tumor vasculature, thereby enhancing immune cell infiltration. Second, certain targeted agents facilitate the conversion of “cold tumors” into “hot tumors”, increasing tumor immunogenicity. Third, targeted therapy can mitigate immunosuppressive pathways, thereby enhancing the efficacy of ICIs. Targeted treatments can alter the TME, making it more conducive to the infiltration and activation of immune cells [145]. In the treatment of HCC, this combination strategy has demonstrated significant advantages. Currently, the predominant combination strategies include anti-angiogenic therapy plus ICIs and TKIs plus ICIs. These regimens not only improve therapeutic outcomes but also expand treatment options for cancer patients.

#### ICIs combined with antiangiogenic drugs

Angiogenesis is a hallmark of HCC progression, with vascular endothelial growth factor (VEGF) and its receptor (VEGFR) playing pivotal roles in regulating both tumor angiogenesis and immunosuppression. VEGF promotes Tregs proliferation, inhibits DCs differentiation and antigen presentation, and enhances the expression of the T cell exhaustion-associated transcription factor TOX, thereby impairing CD8^+^ T cell-mediated antitumor immunity [73, 146]. Among the VEGF family members, vascular endothelial growth factor A (VEGFA) stands out as one of the most crucial ligands, while vascular endothelial growth factor receptor-2 (VEGFR2) is expressed in nearly all endothelial cells. Binding of VEGFA to VEGFR2 triggers a cascade of phosphorylation events, activating downstream PI3K/AKT and rapidly accelerated fibrosarcoma/mitogen-activated protein kinase (RAF/MAPK) pathways, thereby stimulating endothelial cell proliferation and migration, crucial for the formation of new vascular branches necessary for tumor growth and metastasis [147]. The hypoxia associated with neovascularization directly impairs the function of TILs. Hypoxia upregulates several inhibitory signals of antitumor immunity, such as PD-L1, IDO, IL-6, and IL-10 [148], while also inducing Tregs recruitment and the polarization of TAMs toward an M2-like phenotype, further promoting immune evasion [149]. In HCC, the highly vascular nature of the tumor often leads to increased permeability of newly formed blood vessels, creating areas of high interstitial pressure and severe hypoxia or necrosis, thereby promoting tumor progression and angiogenesis [150].

**Fig. 4:**
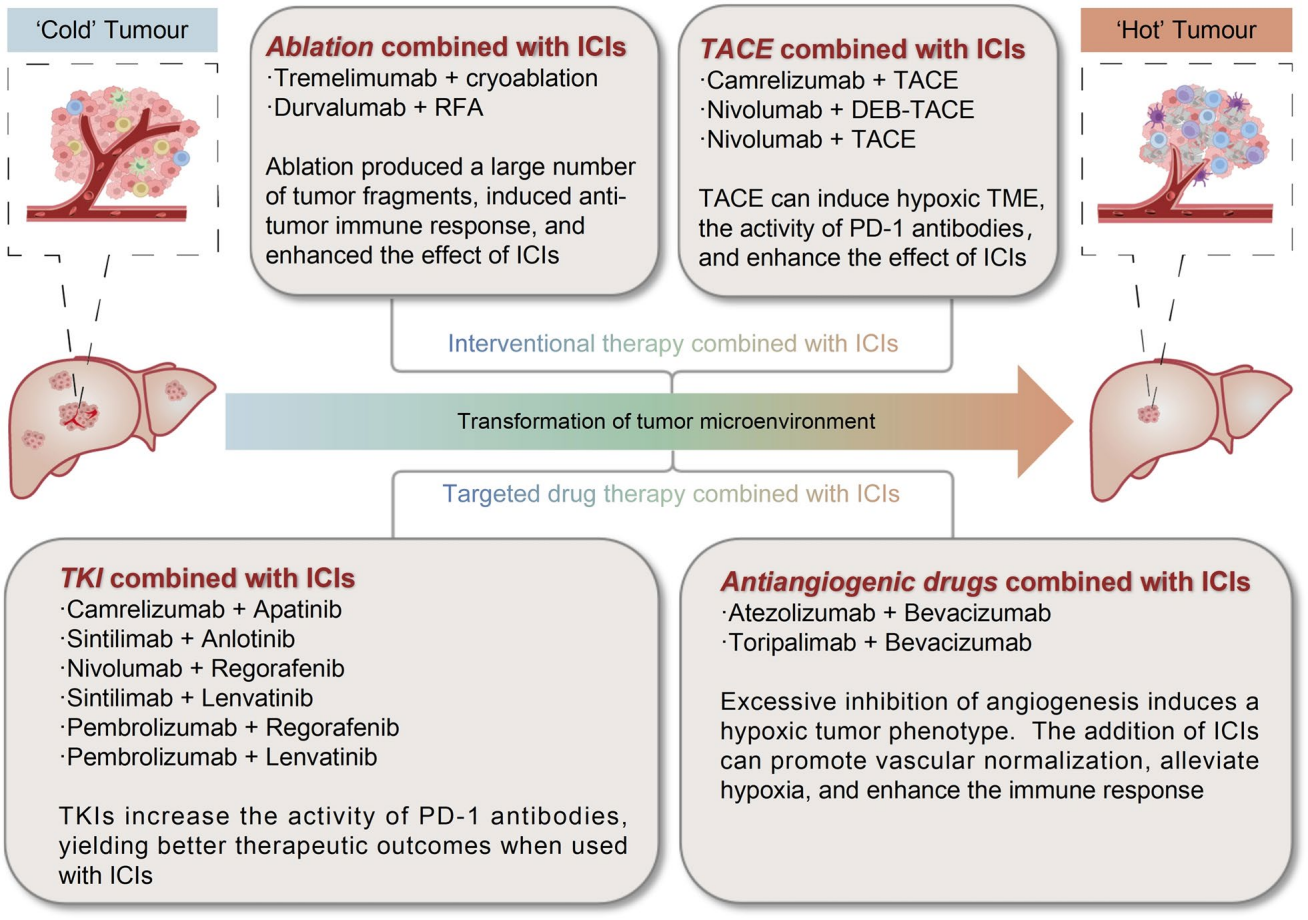
Efficacy of ICI combination therapy. The integration of ICIs with targeted drug therapy or localized treatments facilitates immune cell infiltration into “cold” tumors, thereby converting them into “hot” tumors and subsequently enhancing response rates. Clinical studies of various combination therapies are actively underway. The figure showcases representative ICIs drugs involved in these studies and explains the roles that ICIs play in these combination treatments

The combination of anti-angiogenic agents with ICIs has demonstrated significant synergistic effects in HCC, primarily involving vascular normalization, enhanced immune cell function, and multifaceted regulation of the TME. The combination of VEGFR2 inhibitors and PD-1 inhibitors has been shown to induce vascular normalization in HCC, reducing vascular leakage, improving tumor perfusion, and thereby promoting immune cell infiltration into tumor tissues [151]. Furthermore, anti-VEGF agents can block inhibitory signals that hinder DCs differentiation, decrease the population of MDSCs, and reduce the infiltration and activity of Tregs, thereby reversing the immunosuppressive state of the TME [152]. By alleviating hypoxia within the TME, anti-angiogenic agents enhance T cell functionality and mitigate T cell exhaustion. When used in combination with ICIs, these agents significantly enhance the efficacy of PD-1/PD-L1 inhibitors, overcoming resistance to monotherapy [153] (Fig. 4).

The IMbrave150 trial established the pivotal role of anti-angiogenic agents combined with ICIs in the treatment of HCC. This study demonstrated that patients receiving the combination therapy of Atezolizumab plus Bevacizumab exhibited superior OS (19.2 vs 13.4 months, HR 0.66) and median PFS (6.9 vs 4.3 months, HR 0.65) compared to patients treated with Sorafenib [107]. Building upon this evidence, Fulgenzi and colleagues assessed the prognostic impact of clinical and pathological characteristics on OS and PFS among HCC patients worldwide. The median OS and PFS were 15.7 months (95% CI: 14.5-NE) and 6.9 months (95% CI: 6.1–8.3), respectively. This confirms that the efficacy and safety of Atezolizumab plus Bevacizumab in clinical practice are comparable to the dedicated Phase III IMbrave150 trial findings [154]. Mechanistically, Atezolizumab enhances the recruitment and activation of effector CD8^+^ T cells, DCs, and NK cells while promoting the M1 antitumor macrophage phenotype. Simultaneously, Bevacizumab reduces the infiltration and activity of MDSCs, Tregs and lowers M2 macrophage polarization, thereby collaboratively modulating the tumor immune microenvironment to enhance antitumor activity [155]. Furthermore, a comparative analysis of the IMbrave150, ORIENT-32 and GO30140 trials revealed that Atezolizumab combined with Bevacizumab significantly outperformed Sorafenib monotherapy. Its high ORR (30%) and prolonged PFS (6.8 months) have established it as the standard first-line treatment for HCC.

The combined use of these agents modulates the tumor immune microenvironment, exerting antitumor effects. Various combinations similar to these are also available, such as Ramucirumab, an IgG1 VEGFR2 antagonist, which has been approved for use in HCC based on the Phase III REACH and REACH-2 studies. In the REACH-2 study, HCC patients treated with Ramucirumab exhibited mPFS, progression time, and OS of 1.7 months, 2.8 months, and 8.7 months, respectively, with an ORR of 10.6%, demonstrating the clinical activity and safety of Ramucirumab in advanced HCC patients not treated with Sorafenib [156]. Although Ramucirumab plus Pembrolizumab has been studied in clinical trials for advanced non-small cell lung cancer (NSCLC), its application in HCC has not been established yet [157].

In summary, the combination of anti-angiogenic therapy and ICIs exerts a synergistic antitumor effect through multiple mechanisms. The combination of Atezolizumab and Bevacizumab has become the standard first-line treatment for HCC, significantly improving OS and PFS. However, the use of anti-angiogenic agents and ICIs may be limited in HCC patients with liver cirrhosis or impaired liver function, as studies suggest that Bevacizumab may increase the risk of bleeding and other severe adverse events in patients with poor liver function (Child–Pugh B or C) [158]. The optimal administration sequence of anti-angiogenic agents and ICIs remains unclear and warrants further investigation. Therefore, while combination therapy has significantly prolonged survival in certain patient populations, further research is needed to fully elucidate its potential in combination with ICIs.

#### ICIs combined with TKIs

Multi-target TKIs such as Sorafenib and Lenvatinib have been approved as first-line therapies for advanced liver cancer [159]. TKIs exert their therapeutic effects by blocking multiple signaling pathways, including VEGFR, FGFR, PDGFR, and CSF1R, thereby not only directly inhibiting tumor cell proliferation but also modulating the TME to enhance the antitumor immune response of ICIs [160]. Unlike anti-angiogenic agents that solely target VEGF, multi-target TKIs like Sorafenib have broad target specificity. They can interact with kinases involved in tumor cell signaling pathways, angiogenesis, and apoptosis, thereby inhibiting angiogenesis and tumor growth [161]. Additionally, TKIs also possess immunomodulatory effects. For instance, after Sorafenib therapy, the numbers of ERK^+^Flt-3^+^ Tregs and MDSCs are significantly decreased, contributing to survival benefits [162]. Regorafenib treatment increases tumor infiltration of CD8^+^CXCR3^+^ T cells and inhibits tumor growth by upregulating C-X-C motif chemokine ligand 10 (CXCL10) expression in HCC cells, thereby improving survival rates [163]. Additionally, regorafenib inhibits M2-type polarization of TAMs via suppression of the CSF1R signaling pathway while promoting macrophage polarization towards the M1 phenotype, thereby enhancing antitumor immune responses [164, 165]. Similarly, Lenvatinib targets both VEGFR and FGFR, increasing the number of CD8^+^IFN-γ^+^ T cells by inhibiting the FGFR signaling pathway, thereby enhancing antitumor immunity and the activity of PD-1 antibodies [166]. Collectively, these findings indicate that TKIs can remodel the TME to augment the efficacy of ICIs. However, studies have shown that TKI treatment upregulates CTLA-4 expression in T cells, which, while promoting T cell infiltration into tumors, may also impair their effector function. Notably, simultaneous blockade of CTLA-4 can enhance T cell-mediated antitumor activity and effectively reverse the immunosuppressive ecosystem [167]. This suggests that ICIs may, to some extent, alleviate resistance to TKI therapy. Therefore, the combination of TKIs and ICIs synergistically enhances immune infiltration through multiple mechanisms, improving the immune microenvironment in HCC and significantly boosting antitumor immune responses (Fig. 4).

Apatinib, also known as an anti-angiogenic agent, is also a small molecule multi-target receptor tyrosine kinase inhibitor. By inhibiting the activity of kinases such as VEGFR1, VEGFR2, and VEGFR3, Apatinib exerts its antitumor effects. The combination of Camrelizumab plus Apatinib has been approved as a first-line treatment for unresectable HCC. The CARES-301 study showed that this combination yielded an ORR of 25.4% and a mPFS of 5.6 months [168]. The combination therapy of Cabozantinib with checkpoint inhibitors has demonstrated clinical activity in solid tumors [169]. The COSMIC-312 trial evaluated the efficacy of Cabozantinib in combination with Atezolizumab versus Sorafenib as first-line systemic therapy for advanced HCC [170]. The combination immunotherapy improved RFS (6.8 vs 4.2 months; HR 0.63; 99% CI 0.44–0.91; *P* = 0.0012) but did not improve OS. In contrast, the Phase II RENOBATE trial tested Regorafenib-Nivolumab as first-line treatment for unresectable HCC, achieving an ORR of 31.0%, meeting the primary endpoint. The mPFS was 7.38 months, with a 1-year overall survival rate of 80.5%, although the median OS was not reached [165]. In 2018, based on the non-inferiority results of the REFLECT trial, Lenvatinib was approved as a first-line treatment for advanced HCC, marking a turning point in systemic treatment options for advanced HCC [158]. The combination therapy of Lenvatinib with Pembrolizumab has demonstrated promising clinical activity as a first-line treatment for advanced HCC [171]. Patients received daily oral Lenvatinib and intravenous Pembrolizumab 200 mg on day 1 of a 21-day cycle. The ORR of the combination therapy was 36.0% (95% CI: 26.6%-46.2%), with a mPFS of 8.6 months and a mOS of 22 months. However, the LEAP-002 study evaluated the efficacy of adding Pembrolizumab to Lenvatinib versus Lenvatinib plus placebo as first-line treatment for unresectable HCC. Compared to Lenvatinib plus placebo, Lenvatinib plus Pembrolizumab did not reach prespecified significance in improving OS and PFS (OS 21.2 vs. 19.0 months; HR 0.84; 95% CI 0.71–1.00; *P* = 0.023, mPFS 8.2 vs 8.0 months; HR 0.87; 95% CI 0.73–1.02; *P* = 0.047) [172]. A cross-trial comparison between IMbrave150 and LEAP-002 suggests that the survival outcomes of Atezolizumab plus Bevacizumab and Lenvatinib are comparable. Meanwhile, a real-world data from Western countries provide further insights into the effectiveness of these treatments in patients with advanced HCC. A direct comparison of first-line combination therapy versus Lenvatinib showed that, while PFS and ORR appeared similar, Atezolizumab plus Bevacizumab was potentially associated with improved OS compared to Lenvatinib (19.7 vs 14.4 months; HR 0.72; 95% CI: 0.54–0.95; *P* = 0.021) [173]. Sintilimab, a novel selective anti-PD-1 monoclonal antibody, has demonstrated promising clinical activity in advanced HCC [174]. A Phase II single-arm study (NCT04042805) showed that the ORR of Sintilimab combined with Lenvatinib was 35% [175]. However, Sorafenib combined with Sintilimab provided better PFS than Lenvatinib (8.2 vs 5.2 months; HR 0.55; 95% CI 0.24–1.29; *P* = 0.161) [176]. Additionally, apart from its combination with Lenvatinib, the combination of Sintilimab and Anlotinib has shown promising clinical activity and manageable toxicity in first-line treatment for advanced HCC. It exhibited a better PFS compared to Lenvatinib plus Pembrolizumab, with an immature median PFS of 14.65 months (95% CI: 5.06-not reached) [177].

The combination therapy of these drugs also brings new hope for HCC patients. In first-line treatment, Lenvatinib plus Pembrolizumab and Camrelizumab plus Apatinib are representative combination regimens, particularly suitable for patients with Child–Pugh class A liver function and no contraindications related to portal hypertension. For patients who fail first-line therapy, Regorafenib plus Nivolumab has demonstrated promising efficacy as a second-line treatment, benefiting those who develop resistance or disease progression after initial therapy. However, despite the therapeutic potential of TKI-ICI combinations, several challenges remain, including cumulative toxicity, interpatient variability in response, acquired resistance, and high treatment costs. Future research should focus on optimizing combination strategies, identifying predictive biomarkers for precise patient selection, and conducting long-term follow-up studies to assess the durability and safety of these therapies (Table 2).[107, 141, 165, 170–172, 175, 177–192]

**Table 2:** Outcomes of combination therapy with ICIs for HCC

Clinical trial information		Treatments (trial)	Phase	n	ORR	mPFS (months)	mOS (months)	Treatment-related adverse event rates		Median follow-up (months)
								Grade ≥ 3	Most common grade 3–4	
No controlled trials	NCT03463876 [178]	Camrelizumab + Apatinib (TKI)	II	70 (first-line)	34.3%	5.7	18-month OS rates were 58.1%	78.6%	Hypertension (40%); elevated serum AST (10%) and/or ALT (8.6%) (Grade 3–5)	16.7
				120 (second-line)	22.5%	5.5	18-month OS rates were 56.5%	76.7%	Hypertension (30.8%); elevated serum AST (10.8%) and/or ALT (6.7%) (Grade 3–5)	14.0
	NCT04052152 [177]	Sintilimab + Anlotinib (TKI)	II	20	40.0%	14.65	NR	40.0%	Hypertension (35%); elevated serum AST (35%) and/or ALT (30%)	12
	NCT04310709 [165]	Nivolumab + Regorafenib (TKI)	II	42	31.0%	7.38	NE	24%	Increased AST (4.8%)	11.1
	NCT04042805 [175]	Sintilimab + Lenvatinib (TKI)	II	26	35%	NE	NE	NE	Hypothyroidism (38%); proteinuria (31%) and hypertension (23%)	9
	NCT03347292 [179]	Pembrolizumab + Regorafenib (TKI)	Ib	35 (REG 120 mg)	88% (DCR)	NR	NR	86%	Hypertension (17%); increased AST (23%) and/or ALT (17%)	11.7
				22 (REG 80 mg)	91% (DCR)	NR	NR	50%	Hypertension (9%); increased AST (9%) and/or ALT (9%)	6.9
	NCT04696055 [191]	Pembrolizumab + Regorafenib (Atezolizumab + Bevacizumab-experienced)	II	68	5.9%	2.8	NR	56%	Palmar-plantar erythrodysesthesia syndrome (39%); asthenia (33%); decreased appetite (32%); diarrhea (28%) and hypertension (20%)	7.1
		Pembrolizumab + Regorafenib (ICIs-experienced)		27	11.1%	4.2	NR			
	NCT03006926 [171]	Pembrolizumab + Lenvatinib (TKI)	Ib	104	36.0%	8.6	22	67%	Hypertension (36%); elevated serum AST (20%) and palmar-plantar erythrodysaesthesiav (23%)	10.6
	NCT03841201 [180]	Nivolumab + Lenvatinib (TKI)	II	50	28%	9.0	27.1	59.1%	NR	NR
	NCT04926532 [181]	Toripalimab + Sorafenib (TKI)	II	28	35.7%	4.8	NE	39.3%	NR	14.7
	NCT04605796 [182]	Toripalimab + Bevacizumab (anti-VEGF)	II	52	32.7%	9.9	NE	25.9%	NR	NR
	ChiCTR1900028295 (ALTER-H003) [183]	Toripalimab + Anlotinib (TKI)	II	26	34.6%	10.2	NE	45.2%	Hypertension (9.7%); hand-foot skin reaction (6.5%); arthralgia (6.5%); total bilirubin increased (6.5%) and fatigue (6.5%)	NR
	ChiCTR1900026163 [184]	Camrelizumab + TACE	real-world study	151	25.9%	4.27	14.0	37.14%	Neutropenia (8.57%); increased ALT and AST (13.57%); thrombocytopenia (17.14%) and hyperbilirubinemia (7.14%)	NR
	NCT03143270 [141, 185]	Nivolumab + DEB-TACE	I	19	21%	NR	12-months OS rate was 71%	NR	Fatigue (53%); increased ALT/AST (42%); fever (37%) and pruritis (32%)	NR
	NCT03572582 [186]	Nivolumab + TACE	II	49	71.4%	7.2	28.3	34.7%	NR	20
	NCT04191889 [187]	Camrelizumab + Apatinib (VEGFR-2 inhibitor) + HAIC	II	35	77.1%	10.38	NE	74.3%	Elevated serum AST (28.6%) and/or ALT (20%)	23.10
	NCT04814043 (PLATIC) [192]	Sintilimab + Lenvatinib (TKI) + TACE + HAIC	II	57	77.2%	14.3	NR	64.9%	Elevated serum ALT (36.8%) and abdominal pain (19.3%)	NR
Controlled trials	NCT03755791 (COSMIC-312) [170]	Atezolizumab + Cabozantinib (TKI) vs Sorafenib	III	432 vs 217	47% vs 8%	6.8 vs 4.2	15.4 vs 15.5	18% vs 8%	Hypertension (9% vs 8%); elevated serum AST (9% vs 4%) and palmar-plantar erythrodysaesthesiav (8% vs 8%)	15.8 vs 15.8
	NCT03713593 (LEAP-002) [172]	Lenvatinib (TKI) + Pembrolizumab vs Lenvatinib + Placebo	III	395 vs 399	26.1% vs 17.5%	8.2 vs 8.0	21.2 vs 19.0	63% vs 58%	Hypertension (18% vs 17%); increased AST (7% vs 5%) and/or ALT (5% vs 3%)	32.1 vs 32.1
	NCT03434379 (IMbrave150) [107, 188]	Atezolizumab + Bevacizumab (anti-VEGF) vs Sorafenib	III	336 vs 165	30% vs 11%	6.8 vs 4.3	19.2 vs 13.4	36% vs 46%	Hypertension (15%); elevated serum AST (7%) and/or ALT (4%)	15.6 vs 15.6
	NCT03764293 (CARES-310) [168]	Camrelizumab + Apatinib (TKI) vs Sorafenib	III	272 vs 271	34.3% vs 19%	5.6 vs 3.7	23.8 vs 15.2	24% vs 6%	Hypertension (38% vs 15%); increased AST (17% vs 5%) and increased ALT (13% vs 3%)	7.8 vs 7.8
	NCT03794440 (ORIENT-32) [189]	Sintilimab + IBI305 (Bevacizumab biosimilar) vs Sorafenib	II/III	380 vs 191	NR	4.6 vs 2.8	NE vs 10.4	32% vs 19%	Hypertension (14% vs 6%) and palmar-plantar erythrodysaesthesiasyndrome (0 vs 12%)	10 vs 10
	NCT02715531 (GO30140) [190]	Atezolizumab + Bevacizumab (anti-VEGF) vs Atezolizumab	Ib	60 vs 59	NR	5.6 vs 3.4	NR	12% vs 3%	Hypertension (5% vs none) and proteinuria (3% vs none)	6.6 vs 6.7

*ALT* alanine aminotransferase, *AST* aspartate aminotransferase, *DOR* uration of response, *HAIC* hepatic artery infusion chemotherapy, *mOS* median overall survival, *mPFS* median progression-free survival, *n* number of patients, *NE* not evaluable, *NR* not reported, *ORR* objective response rate, *RFS* recurrence-free survival, *TACE* transcatheter arterial chemoembolization, *TKI* tyrosine kinase inhibitors, *TTP* time to progression

### ICIs combined with multiple conventional therapies

The potential combination therapies for HCC include, but are not limited to, the integration of chemotherapy, targeted therapy, radiation therapy, and even local ablation methods. For instance, chemotherapy not only directly eradicates tumor cells but also induces immunogenic cell death (ICD), thereby promoting the release of tumor antigens and enhancing antigen presentation, ultimately improving the efficacy of ICIs [193, 194]. Moreover, certain chemotherapeutic agents can suppress immunosuppressive cells such as MDSCs and Tregs, further modulating the TME in favor of anti-tumor immunity [195]. In addition to its direct cytotoxic effects, radiotherapy can also trigger the “abscopal effect”, leading to the release of tumor-associated antigens and upregulation of MHC-I molecules, which enhances antigen presentation and augments ICI-mediated T cell cytotoxicity [196]. Furthermore, radiotherapy has been shown to reduce the expression of immunosuppressive molecules such as TGF-β and IL-10, thereby improving the immunosuppressive TME and facilitating a more effective ICI response [197]. Given these mechanisms, the combination of three or more treatments has become a common strategy for the treatment of HCC (Fig. 2).

A multimodal comprehensive treatment strategy provides a promising avenue for tumor conversion therapy, enabling patients with unresectable advanced HCC to regain the opportunity for surgical resection while effectively reducing postoperative recurrence and metastasis rates, ultimately leading to long-term survival benefits. In a nationwide retrospective cohort study, HCC patients underwent either TACE with PD-1/PD-L1 inhibitors plus MTT (TKI or anti-VEGF monoclonal antibody) or TACE monotherapy. The findings indicated significant improvements in PFS, OS, and ORR among the combination therapy group in Chinese patients, with an ORR of 60.1% compared to 32.0% (*P* < 0.001), PFS of 9.5 vs 8.0 months (HR, 0.70, *P* = 0.002), and OS of 19.2 vs 15.7 months (HR, 0.63, *P* = 0.001) [139]. In another study, Long et al. conducted a retrospective investigation on the triple combination therapy of TACE or hepatic artery infusion chemotherapy (HAIC) combined with TKIs and ICIs, revealing an encouraging ORR of approximately 60%, along with a conversion surgery rate of 20–40% [198]. Additionally, Zhang et al. evaluated the efficacy of Camrelizumab and Apatinib combined with HAIC-FOLFOX (Oxaliplatin, Fluorouracil, and Leucovorin) in treating BCLC-C stage HCC patients, reporting an ORR of 77.1% (95% CI: 59.9%-89.6%) and a median PFS time of 10.38 months (95% CI: 7.79–12.45). Notably, compared to the TACE group, the cure rate of HAIC-FOLFOX surgery increased to 24%, significantly higher than the 12% observed in the TACE monotherapy group, with overall improvement in patients’ health status and significant symptom relief [187]. Encouragingly, the Phase II PLATIC study demonstrated that with a median of only three treatment cycles, the quadruple regimen of TACE, HAIC, Sintilimab, and Lenvatinib achieved an impressive conversion resection rate of 77.2%, with a pathological complete response (pCR) rate of 29.5% among resected patients. It is much higher than HAIC-FOLFOX + Camrelizumab + Apatinib (24%), which opens up a new treatment idea for the conversion therapy of unresectable HCC [192]. In patients with advanced HCC, symptomatic portal hypertension (SPH) often occurs as a complication, manifested by variceal bleeding and refractory ascites, which can limit the efficacy of ICIs and certain targeted therapies. However, transjugular intrahepatic portosystemic shunt (TIPS) directly alleviates portal hypertension, thereby providing an opportunity for systemic treatment in patients with advanced HCC complicated by SPH [199]. A study has assessed the efficacy of TIPS combined with Lenvatinib and PD-1 inhibitors in advanced HCC patients with SPH, demonstrating a lower recurrence rate of SPH and acceptable side effects (ORR 38.5%; 95% CI 11.8%-61.6%, OS 16.5 months; 95% CI 16.1–17.0) [200]. Li et al. found that dual blockade of PD-L1 and VEGFA (DPVB) combined with low-dose radiotherapy (LDRT) induces rapid tumor inflammation, rendering it susceptible to immunotherapy. Their study elucidates that LDRT recruits stem cell-like progenitor exhausted CD8^+^ T cells (CD8^+^ Tpex) from draining lymph nodes to the tumor site via the CXCL10/CXCR3 axis, enhancing the sensitivity of tumors to DPVB and achieving tumor regression [201].

Currently, in the field of HCC treatment, the combination therapy of ICIs with targeted therapies and TACE is extensively studied, presenting a rich research landscape (Fig. 4). However, compared to this, clinical research on the combination of radiotherapy and ICIs in treating HCC is relatively scarce. Nevertheless, existing studies have demonstrated that radiotherapy can facilitate the effect of ICIs in creating a more inflammatory-sensitive microenvironment. Therefore, future clinical research is expected to shift its focus towards the combined application of radiotherapy and ICIs, and explore optimal radiotherapy dosage strategies. With further research advancement, the combined use of various treatment modalities is anticipated to become more flexible, offering greater potential for the role of ICIs in HCC treatment.

## Potential clinical combinations of ICIs and novel materials in HCC

In recent years, various materials have played a crucial role in synergizing with ICIs to exert antitumor effects. These materials not only facilitate the efficient delivery of ICIs and reverse the immunosuppressive TME, but also work in conjunction with therapies such as chemotherapy and ablation to inhibit tumor growth and metastasis. Additionally, these materials can induce ICD in tumor cells, thereby effectively enhancing immune responses. This section explores the mechanisms and clinical potential of these novel materials in combination with ICIs.

In the context of tumor immunosuppression, the efficient delivery of ICIs is critical to overcoming tumor resistance, immune tolerance, and irAEs. For example, FDA-approved MRI-visible magnetic nanoparticles (ferumoxytol; Fer) can bind to PD-L1 antibodies via Z-linkers, enabling targeted delivery of ICI monoclonal antibodies (mAbs), thus maximizing the PD-L1 blockade efficiency in treating HCC [202]. In addition to targeted delivery, these magnetic nanoparticles can also assist in magnetic thermal ablation. The magnetic thermal ablation of PAM@Fe_3_O_4_ MSs can activate the immune system, promote immune cell infiltration into the tumor, and work synergistically with PD-1 mAbs to exert antitumor effects. PAM@Fe_3_O_4_ MSs possess excellent biocompatibility and outstanding eddy current heating effects. Under high-frequency alternating magnetic fields, their combination with PD-1 mAbs stimulates increased infiltration of CD3^+^ T cells into the tumor, thereby enhancing immune responses [203]. However, a major obstacle in combining ICIs with thermal ablation is insufficient antigen internalization and the immaturity of tumor-infiltrating dendritic cells (TIDCs), which result in a poor immune response to distant tumor growth. By co-delivering tumor-associated antigens and fat mass and obesity associated gene inhibitors to TIDCs, this approach aims to increase m6A methylation, promote dendritic cell maturation, and activate antitumor immunity following HCC thermal ablation, thereby assisting ICI in suppressing distant tumor growth and metastasis [204]. Moreover, the enhanced glycolytic activity of residual HCC cells after ablation leads to an accumulation of lactate in the tumor, which may further exacerbate the immunosuppressive TME. LOX-MnO_2_@Gel-mediated local lactate depletion can convert the immunosuppressive post-ablation TME into an immune-active environment, and when combined with ICIs therapy, it significantly inhibits residual HCC growth and lung metastasis, thereby extending the survival of mice post-ablation [205].

M2 macrophages play a crucial role in the immunosuppressive TME, where they are typically associated with immune suppression and pro-tumor activity. M2 macrophages can directly interact with PD-1 on T cells by expressing immune checkpoint molecules such as PD-L1, thereby inhibiting T cell proliferation and activation, leading to immune tolerance. Promoting the polarization of M2 macrophages toward the M1 phenotype through drugs or nanoparticles can enhance the efficacy of immunotherapeutic agents. During the progression of HCC, CCL2 and CCL5 have been shown to play key roles in the M2 polarization of TAMs. Delivering mRNA encoding BisCCL2/5i can achieve dual inhibition of these two molecules, resulting in the polarization of macrophages toward the M1 phenotype and reducing the immunosuppressive effects in the TME [206]. Furthermore, combining this approach with PD-L1 inhibitors can significantly prolong survival and promote the eradication of diffuse liver cancer. Similarly, the selective BCL-2 inhibitor APG-2575 can polarize immunosuppressive M2 macrophages into M1 immunostimulatory macrophages, thereby enhancing T cell function and synergizing with PD-1 inhibitor therapy [207]. Additionally, p53 activation can suppress M2 macrophage polarization. The MDM2 antagonist APG-115, a pharmacological activator of p53, has been shown to enhance the antitumor immune response induced by PD-1 therapy, thereby inhibiting tumor progression [208].

Moreover, inducing ICD in cancer cells has emerged as a highly promising strategy for tumor therapy. Under external or endogenous stimuli, certain apoptotic tumor cells can release immunogenic proteins, thereby triggering an antitumor immune response. In this process, CTLs are activated to more effectively eliminate tumor cells, ultimately achieving an enhanced antitumor effect. This phenomenon is referred to as tumor cell ICD [209]. Chemo-photodynamic therapy (chemo-PDT) can elicit antitumor immune responses and synergize with PD-L1 antibodies to exert distant effects, inhibiting both primary and metastatic tumor growth. For example, TB/PTX@RTK micelles actively target tumor cells, and upon light irradiation, paclitaxel (PTX) is released, inducing ICD and releasing tumor-associated antigens (TAA) to promote DC maturation and cytotoxic T cell activation. At the same time, chemotherapy and photodynamic therapy upregulate PD-L1 expression in tumor cells, reversing the immunosuppressive TME [210]. Additionally, using chemo-PDT with ROS-sensitive lipid-polymer hybrid nanoparticles (TKHNP-C/D) enhances the antitumor efficacy of anti-PD-L1 antibodies. Upon light irradiation, these nanoparticles not only induce PDT but also promote drug release through rapid degradation of their core, effectively inhibiting tumor growth with a single treatment [211].

To conclude, various novel materials play a critical role in synergistic ICI immunotherapy through different mechanisms (Table 3). These materials not only enhance the immunogenicity of tumors but also improve the immune microenvironment, thereby effectively boosting antitumor efficacy. Future research should focus on optimizing material design, exploring combination strategies, and conducting clinical trials to validate these approaches. By integrating novel materials with ICIs, we can unlock new possibilities for HCC treatment, ultimately improving patient outcomes.

**Table 3:** Mechanisms of action of materials combined with ICIs

Material name	Function of materials	Associative mechanism	References
aPD-L1-Z-Fer	Enhancing drug delivery efficiency	The Z protein chain on the Fer surface enables the targeted binding of aPD-L1, exposing the active Fab binding site and effectively blocking immune checkpoints	[202]
P/LHNVs (Cationic Polymer-Lipid-Hybrid Nanovesicles)	Enhancing drug delivery efficiency	(1) Using cationic nanoparticles as carriers, they effectively deliver immunostimulatory agents like siRNA to tumor sites (2) Immunostimulatory modifications induce tumor cells to release damage-associated molecular patterns (DAMPs), thereby activating the immune system (3) siRNA-mediated knockdown of PD-L1 expression reduces the presence of PD-L1 on tumor cell surfaces, inhibiting their immune evasion mechanisms The synergistic action of these two drug types stimulates the body’s immune system to initiate antitumor responses, thereby inhibiting tumor growth	[217]
PAM@Fe_3_O_4_ microspheres	Heat ablation	PAM@Fe_3_O_4_-MSs, along with MTA, can activate the immune system of the host, promoting immune cell infiltration into tumor tissues. Combined with PD-1 mAb, they synergistically enhance the antitumor effect	[203]
M/m-MP@F (Man/mal-MPDA@ FB 23–2)	Heat ablation	TAA and the FTO inhibitor are co-delivered to TIDCs, which is expected to trigger a robust antitumor immune response by increasing m6A methylation, promoting DCs maturation, and activating antitumor immunity after HCC ablation. This process supports ICI by inhibiting the growth and metastasis of distant tumors	[204]
LOX-MnO_2_@Gel Injectable Hydrogel System	Reversing immunosuppressive microenvironment	The LOX-MnO_2_@Gel system generates oxygen to relieve hypoxia in the TME, transforming the TME into a more immunostimulatory state. This system synergizes with ICI therapy to improve antitumor immune responses, slowing tumor progression and reducing metastasis	[205]
BisCCL2/5i-mRNA Nanoplatform	Reversing immunosuppressive microenvironment	The BisCCL2/5i-mRNA nanoplatform targets the tumor microenvironment, reprogramming it to enhance immune infiltration, and synergizes with ICIs for cancer therapy	[206]
APG-2575	Reversing immunosuppressive microenvironment	APG-2575 inhibits BCL-2 expression, reducing the number of MDSCs and activating the NLRP3 inflammasome, which improves the efficacy of anti-PD-1 therapy	[207]
APG-115	Reversing immunosuppressive microenvironment	APG-115 activates p53 to modulate the TME, enhancing the efficacy of ICIs	[208]
BPSP (BP/SF/anti-PD-L1 mAb)	Reversing immunosuppressive microenvironment	(1) NIR-II-triggered PTT mediated by BPSP remodels the ECM, which facilitates immune cell infiltration and alleviates immunosuppression (2) The introduction of SF suppresses the RAS/RAF/ERK pathway to downregulate PD-L1 expression (3) Anti-PD-L1 mAb blocks PD-1/PD-L1 signaling to promote tumor cell death, and PTT-mediated ICD further enhances the effect of the anti-PD-L1 antibody	[218]
CAT@CaCO_3_	Reversing immunosuppressive microenvironment	The release of Ca^2^⁺ by tumor cells leads to intracellular Ca^2^⁺ overload, which triggers the release of DAMPs signals and initiates an antitumor immune response. Meanwhile, the immunostimulatory TME induced by CAT@CaCO_3_ nanoparticles promotes the polarization of M2 tumor-associated macrophages to the M1 phenotype, further enhancing tumor antigen presentation. Consequently, T cell-mediated antitumor responses are activated, and their efficacy is further boosted by aPD-1 immune checkpoint blockade	[219]
MAL-NPs	Reversing immunosuppressive microenvironment	(1) RT induces antigen release; (2) MAL-NPs capture antigens and accumulate at the tumor site; (3) RT reduces R848-N3, releasing R848; (4) The RT-induced reduction of R848 stimulates DCs activation, enhancing antigen presentation by DCs; (5) DCs migrate to lymph nodes and present tumor antigens to naïve CD8⁺ T cells, activating T cells and inducing tumor-specific T cell cytotoxicity, thereby synergistically enhancing the efficacy of ICIs	[220]
TB/PTX@RTK (AIE, TB and PTX-Loaded Nanoparticles)	Inducing ICD in tumor cells	Under NIR irradiation, the photothermal effect of TB produces ROS, and the chemotherapeutic agent PTX inhibits tumor growth, triggering ICD and releasing tumor antigens for immune activation. This enhances immune cell infiltration into the tumor	[210]
^TK^HNP-C/D (ROS-Sensitive Prodrug Nanoparticles Loaded)	Inducing ICD in tumor cells	Under 660 nm laser irradiation, ^TK^HNP-C/D produces ROS, which enhances the phototoxic effect of the PDT and induces the immunogenic death of tumor cells. The ROS also activates DCs, thereby enhancing the immune response and assisting in the regulation of the PD-L1 immune pathway	[211]
Gep and AL Nanocomposite Sheet	Inducing ICD in tumor cells	The application of AL under NIR light leads to local vascular permeability and immune cell infiltration, promoting the immunogenic death of tumor cells. This enhances the penetration of immune cells through the vascular walls, increasing cytokine secretion, and upregulating the PD-L1 antibody response	[221]
MDP NPs (MnO_2_/DOX metal-polyphenols Nanoparticles)	Inducing ICD in tumor cells	MDP-NPs generate ROS and release proinflammatory cytokines, which activate DCs and macrophages in the tumor microenvironment. Through these mechanisms, MDP-NPs stimulate a strong immune response that synergizes with ICI therapy, enhancing antitumor effects	[222]

*AIE*: aggregation induced emission, *AL* anlotinib, *DAMPs* damage-associated molecular patterns, DOX, *FTO* fat mass and obesity associated gene, *Gep* Germanium phosphorus, *MDSCs* myeloid-derived suppressor cells, *MTA* magnetic thermal ablation, *PDT* photodynamic therapy, *PTT* performs photothermal therapy, *PTX* paclitaxel, *ROS* reactive oxygen species, *RT* radiotherapy, *SF* silk fibroin, *TAA* tumor-associated antigens, *TB* photosensitizer, *TIDCs* tumor-infiltrating dendritic cells

## Conclusions

**Fig. 5:**
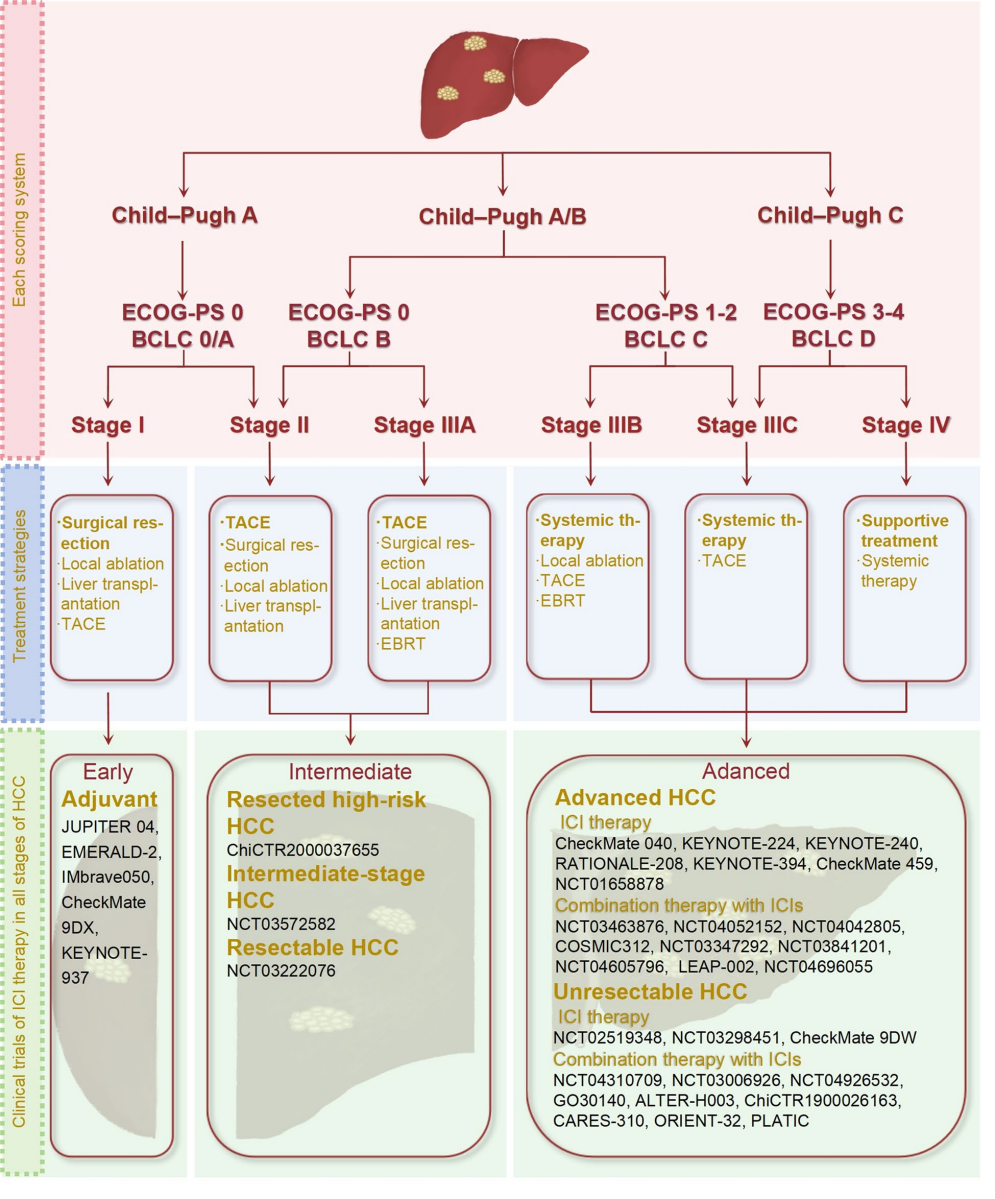
Treatment of HCC at different stages. There are various staging standards for HCC, such as the Child–Pugh classification for liver function, the Barcelona Clinic Liver Cancer (BCLC) staging system, and the Eastern Cooperative Oncology Group (ECOG) performance status. The relationships among these staging systems are illustrated in the figure. The TNM staging system categorizes HCC into six stages based on the severity and progression of the disease, with each stage associated with distinct therapeutic strategies. The primary therapeutic strategy for each stage is highlighted in bold in the figure. Although there are numerous staging systems for HCC, from a developmental perspective, liver cancer can be broadly categorized into early, intermediate, and advanced stages. Currently, ICI have been applied in the treatment of HCC across all these stages. Furthermore, relevant clinical trials have been shown in the figure

As ICI therapy progresses, treatment strategies centered around it are poised to significantly alter the future landscape of HCC therapy. Currently, the combination of ICIs with other therapeutic modalities has emerged as a novel trend in HCC treatment, with the most prevalent regimens encompassing dual immunotherapy, combined anti-angiogenic agents, and amalgamated radiotherapy with chemotherapy (Fig. 5). Notably, combining multiple ICIs with various other therapeutic approaches, including multifunctional materials for enhanced delivery and targeting, may offer effective routes for HCC treatment. However, tailoring specific combinations to individual patients requires further research to optimize efficacy and safety. Multiple clinical studies have underscored that compared to monotherapy with Sorafenib, ICIs combination therapy substantially prolongs patients’ survival. Furthermore, materials designed for targeted delivery and controlled release enhance the efficacy and safety of ICIs, facilitating improved therapeutic outcomes in HCC. These advancements underscore the transformative potential of ICI combination therapy over monotherapy, significantly prolonging patient survival. Nonetheless, the integration of ICIs in treatment continues to confront numerous challenges. Further study and refinement are needed to determine the optimal combination therapies for HCC patients at different stages, including establishing the best timing for combination therapy, optimizing the integration of immune checkpoint inhibitors with other therapeutic modalities, and effectively mitigating treatment-related adverse events.

Currently, combination therapies involving targeted therapy and ICIs, such as Atezolizumab plus Bevacizumab, Sintilimab plus a Bevacizumab biosimilar, and Camrelizumab plus Apatinib, as well as dual immunotherapy regimens like Durvalumab plus Tremelimumab, have been recommended as first-line treatment options for advanced HCC. With targeted-ICI and dual-ICI regimens becoming standard first-line therapies for advanced HCC, an increasing number of patients will experience disease progression following ICI-based treatment. However, there is currently no established second-line treatment recommendation for ICI-refractory HCC, and high-level evidence remains lacking. Following the failure of first-line immunotherapy combinations, treatment selection should be tailored based on the pattern of disease progression and the specific first-line regimen used. It is crucial to consider the anticancer mechanisms, clinical efficacy, safety, and tolerability of second-line options to formulate an optimal treatment strategy.

Future research should prioritize a comprehensive investigation into the TME and the molecular mechanisms underlying immune checkpoint modulation, which will facilitate the identification of novel ICI targets. A critical focus should also be placed on discovering predictive biomarkers for ICI efficacy and tolerance, including circulating tumor DNA (ctDNA), TMB, TME characteristics, and microbiome profiles. Advanced multi-omics approaches, such as transcriptomics, proteomics, and metabolomics, should be utilized to identify HCC-specific biomarkers associated with immunotherapy responses. Large-scale cohort studies and retrospective analyses are essential to validate the correlation between these biomarkers and ICI treatment outcomes.

Furthermore, research efforts should aim to optimize the sequencing and combination of ICIs with other therapeutic modalities, such as TACE, HAIC, radiotherapy, and targeted therapies, to determine the most effective treatment timing and strategy. For instance, short-term intervention trials could evaluate the administration of ICIs prior to surgery, assessing their impact on the TME and identifying patients who are likely to benefit from such approaches. Randomized controlled trials comparing different treatment sequences, such as ICI-first versus targeted therapy-first versus concurrent combination, are also necessary to establish optimal therapeutic protocols.

To address the challenge of ICI resistance, future studies should investigate its molecular basis, including the roles of immunosuppressive cells such as Tregs and MDSCs, metabolic reprogramming, and tumor immune evasion mechanisms. Strategies to overcome resistance, such as targeting metabolic pathways, combining ICIs with radiotherapy, or modulating novel co-stimulatory and co-inhibitory pathways including TIGIT, LAG-3 and CD47, warrant further exploration.

Finally, optimizing the management of irAEs is of paramount importance. Research should focus on identifying biomarkers predictive of irAEs and developing personalized immune management strategies. Prospective studies comparing different approaches to managing irAEs are needed to enhance the safety and adherence of ICI therapy. Although ICI therapy holds promise in overcoming acquired resistance and reducing the incidence of irAEs, determining the optimal combination strategies for different stages of HCC remains a significant challenge.

In summary, ICI therapy has revolutionized the treatment landscape for HCC, offering new hope for patients. Ongoing research continues to refine therapeutic paradigms, with the ultimate goal of improving clinical outcomes and enhancing the quality of life for patients.

## Data Availability

No datasets were generated or analysed during the current study.

## Abbreviations

ALT: Alanine aminotransferase
APCs: Antigen-presenting cells
Arg-1: Arginase-1
AST: Aspartate aminotransferase
BCR: B cell receptor
BTLA: B and T lymphocyte attenuator
chemo-PDT: Chemo-photodynamic therapy
ctDNA: Circulating tumor DNA
CTLA-4: Cytotoxic T-lymphocyte antigen 4
DCs: Dendritic cells
DOR: Duration of response
Gal-9: Galectin-9
HAIC: Hepatic artery infusion chemotherapy
HCC: Hepatocellular carcinomam
HR: Hazard ratio
HDAC3: Histone deacetylase 3
HVEM: Herpesvirus entry mediator
ICD: Immunogenic cell death
IDO: Indoleamine 2,3-dioxygenase
IGSF8: Immunoglobulin superfamily member 8
IL-2: Interleukin-2
iNOS: Inducible nitric oxide synthase
irAEs: Immune-related adverse events
ITPRIPL1: Inositol 1,4,5-trisphosphate receptor interacting protein like 1
LAG-3: Lymphocyte-activation gene 3
LDRT: Low-dose radiotherapy
LIHC: Liver hepatocellular carcinoma
MDSCs: Myeloid-derived suppressor cells
MHC: Major histocompatibility complex
mPFS: Median progression-free survival
n: Number of patients
NE: Not evaluable
NK: Natural killer
NKG2A: Natural killer group 2A
NKT: Natural killer T
NLRP3: NOD-like receptor family pyrin domain containing 3
NR: Not reported
ORR: Objective response rate
OS: Median overall survival
pCD: Pathological complete response
PD-1: Programmed cell death 1
PD-L1: Programmed cell death ligand 1
PFS: Progression-free survival
PI3K/AKT: Phosphatidylinositol 3 kinase/protein kinase B
PSGL-1: P-selectin glycoprotein ligand 1
PTX: Paclitaxe
RAF/MAPK: Rapidly accelerated fibrosarcoma/mitogen-activated protein kinase
RFA: Radiofrequency ablation
RFS: Recurrence-free survival
RGMb: Repulsive guidance molecule B
ROS: Reactive oxygen species
SPH: Symptomatic portal hypertension
STING: Stimulator of interferon genes
TAA: Tumor-associated antigens
TACE: Transarterial chemoembolization
TCR: T cell receptor
TGF-β: Transforming growth factor-β
TIDCs: Tumor-infiltrating dendritic cells
TIGIT: T cell immunoreceptor with Ig and ITIM domains
TILs: Tumor-infiltrating lymphocytes
TIM-3: T cell immunoglobulin and mucin-domain containing 3
TIPS: Transjugular intrahepatic portosystemic shunt
TKIs: Tyrosine kinase inhibitors
TMB: Tumor mutational burden
TME: Tumor microenvironment
Tregs: Regulatory T cells
TTP: Time to progression
VEGF: Vascular endothelial growth factor
VISTA: V-domain Ig suppressor of T cell activation
